# AAA + ATPase Thorase inhibits mTOR signaling through the disassembly of the mTOR complex 1

**DOI:** 10.1038/s41467-022-32365-2

**Published:** 2022-08-17

**Authors:** George K. E. Umanah, Leire Abalde-Atristain, Mohammed Repon Khan, Jaba Mitra, Mohamad Aasif Dar, Melissa Chang, Kavya Tangella, Amy McNamara, Samuel Bennett, Rong Chen, Vasudha Aggarwal, Marisol Cortes, Paul F. Worley, Taekjip Ha, Ted M. Dawson, Valina L. Dawson

**Affiliations:** 1 Neuroregeneration and Stem Cell Programs, Institute for Cell Engineering, Johns Hopkins University School of Medicine, Baltimore, MD 21205 USA; 2 Department of Neurology, Johns Hopkins University School of Medicine, Baltimore, MD 21205 USA; 3 Cellular and Molecular Medicine Graduate Program, Johns Hopkins University School of Medicine, Baltimore, MD 21205 USA; 4 Department of Materials Science and Engineering, University of Illinois at Urbana-Champaign, Urbana, IL 61801 USA; 5 Department of Biophysics and Biophysical Chemistry, Johns Hopkins University, Baltimore, MD 21205 USA; 6 Solomon H. Snyder Department of Neuroscience, Johns Hopkins University School of Medicine, Baltimore, MD 21205 USA; 7 Departments of Biophysics and Biophysical Chemistry, Biophysics and Biomedical Engineering, JHU Howard Hughes Medical Institute, Baltimore, MD 21205 USA; 8 Department of Pharmacology and Molecular Sciences, Johns Hopkins University School of Medicine, Baltimore, MD 21205 USA; 9 Department of Physiology, Johns Hopkins University School of Medicine, Baltimore, MD 21205 USA; 10 Present Address: Division of Neuroscience, National Institute of Neurological Disorders and Stroke, National Institutes of Health, Bethesda, MD USA; 11 Present Address: Vollum Institute, Oregon Health & Science University, Portland, OR 97239 USA

**Keywords:** Cell signalling, Lysosomes, Mechanisms of disease

## Abstract

The mechanistic target of rapamycin (mTOR) signals through the mTOR complex 1 (mTORC1) and the mTOR complex 2 to maintain cellular and organismal homeostasis. Failure to finely tune mTOR activity results in metabolic dysregulation and disease. While there is substantial understanding of the molecular events leading mTORC1 activation at the lysosome, remarkably little is known about what terminates mTORC1 signaling. Here, we show that the AAA + ATPase Thorase directly binds mTOR, thereby orchestrating the disassembly and inactivation of mTORC1. Thorase disrupts the association of mTOR to Raptor at the mitochondria-lysosome interface and this action is sensitive to amino acids. Lack of Thorase causes accumulation of mTOR-Raptor complexes and altered mTORC1 disassembly/re-assembly dynamics upon changes in amino acid availability. The resulting excessive mTORC1 can be counteracted with rapamycin in vitro and in vivo. Collectively, we reveal Thorase as a key component of the mTOR pathway that disassembles and thus inhibits mTORC1.

## Abstract (web summary)

Signaling via the mTOR complex 1 (mTORC1) maintains cellular and organismal homeostasis. Here the authors show that the AAA + ATPase Thorase binds mTOR to promote disassembly and inactivation of mTORC1 to fine tune TOR signaling according to amino acid availability.

## Introduction

The mechanistic target of rapamycin (mTOR) is a widely conserved protein kinase that governs the growth and survival of cells through two functionally distinct multiprotein complexes, the mTOR complex 1 (mTORC1) and the mTOR complex 2^1,2^. The mTORC1 senses and integrates information about nutrient and growth factor availability, cellular energy levels, and stress to coordinate growth by acting as a switch between synthetic and degradative metabolic processes^3,4^. Deregulation of the mTOR signaling pathway underpins numerous human diseases, spanning from cancer and diabetes to diverse neurological disorders^1,2^.

Given the central role of mTOR in organismal survival, extensive efforts have been directed towards understanding how this pathway is regulated. Considerable advances have been made to elucidate the mechanisms leading to the activation of mTORC1^3–7^. All upstream cues that inform mTOR on the energy, nutrient, and stress status of the cell funnel through two sets of small G proteins, the Rheb GTPase and the heterodimeric A-D Rag GTPases^8^. Those cues are sensed by a wide array of proteins that alter the nucleotide binding status (GTP vs GDP) of Rheb and Rags, thereby dictating whether they are “on” to stimulate mTORC1 signaling^8^. Raptor (regulatory-associated protein of mTOR) can grasp the “on” state of the Rag proteins (RagA/B bound to GTP, and RagC/D to GDP)^9^ and interact with them to recruit mTORC1 to the lysosome^6,7^, where GTP-Rheb can stimulate the kinase activity of mTOR^10,11^. This ensures mTORC1 signaling occurs only if the conditions are optimal to grow (upon coincidence detection of growth factors and nutrients, while devoid of cellular stress). While mTOR association with Raptor is critical for mTORC1 subcellular localization and substrate recruitment^12^, if and how mTOR and Raptor disassociate remains unclear.

In this study, we uncovered a novel role for AAA + ATPase Thorase in the disassembly and inactivation of the mTORC1. Thorase, also known as ATPase family AAA domain-containing protein 1 (ATAD1), uses the energy garnered from ATP hydrolysis to disassemble protein complexes^13,14^, with roles in endocytosis and internalization of α-amino-3-hydroxy-5-methyl-4-isoxazolepropionic acid (AMPA) receptors^14–16^, maintenance of mitochondrial and peroxisomal integrity^17–19^, and an emerging involvement in human disease^16,20,21^. Thorase anchors to the outer mitochondrial membrane through its N-terminal transmembrane domain^17–19^, but its role at the mitochondria is not fully known. Here, we provide evidence that Thorase directly binds mTOR, thereby blocking the binding of mTOR to Raptor and other mTORC1 components. We detect Thorase-mTOR interactions at the interface between mitochondria and lysosomes, where Thorase predominantly exists within the cell and where mTOR must be recruited for activation, respectively. Consistent with mTORC1 signaling responding to nutrient availability, Thorase disassembly of mTORC1 is sensitive to amino acids. When Thorase is lacking, we show that mTORC1 disassociation is decreased resulting in upregulation of mTORC1 signaling in vitro and in vivo, and we can counteract this excessive activity with the mTOR inhibitor, rapamycin. Collectively, our findings reveal a key role for Thorase as inhibitor of mTORC1 signaling that acts through the disassembly of the mTORC1.

## Results

### Thorase-mTOR binding correlates with less mTORC1 protein interaction

**Fig. 1:**
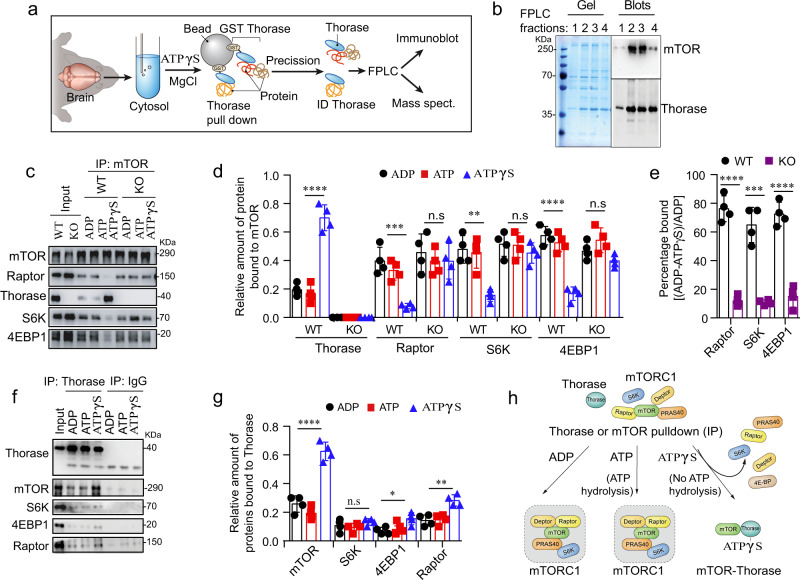
Thorase interaction with mTOR is concomitant to a decrease in binding of mTOR to other mTOR complex 1-related proteins. **a** Schematic diagram of recombinant Thorase pulldown from brain lysates to identify novel binding partners through mass spectrometry. **b** SDS-PAGE and immunoblots of FPLC fractions of Thorase pulldown of mTOR. **c** Immunoblot images of mTOR pulldown from wild type (WT) or Thorase knockout (KO) brain lysates in the presence of different nucleotides. **d** Quantification of blots in **c** (*n* = 4 independent pull-down assays). **e** Graphical representation of percent disassembly of mTORC1-related proteins in **c** (*n* = 4 independent pull-down assays). **f** Immunoblot images of immunoprecipitation (IP) of Thorase in the presence of different nucleotides to pulldown mTORC1-associated proteins. **g** Graphical representation of quantified blots in **f** (*n* = 4 independent pull-down assays). **h** Schematic diagram of Thorase interaction with mTORC1 in the presence of different nucleotides. Data in **d**, **e**, and **g** are mean ± standard error of the mean [SEM] of four independent experiments, ***p < 0.001, *p < 0.05, n.s *p* > 0.05; Data in **d** and **g** are analyzed by one-way ANOVA with Tukey’s post-hoc test, and data (**e**) analyzed by two-tailed unpaired *t*-test (exact *p* values indicated in Data Source File). mTOR peptides from Replicate 1 (out of three replicates) and list of Replicate 1 hits shown in Supplementary Table 1.

In search for Thorase-binding cytosolic proteins that could point us towards new Thorase functions, we designed a method to identify proteins that specifically interact with the active, ATP-bound, form of Thorase^14,16^ (Fig. 1a). We constructed a recombinant Thorase protein with a GST tag that could be cleaved via a protease prescission cleavage site between GST and Thorase. Performing Thorase immunoprecipitations in the presence of a non-hydrolyzable ATP analog (ATPγS) mimics the active form of Thorase when it binds to its substrates^14,16^. We thus mixed GST-Thorase purified on beads with mouse whole brain cytosolic extract in the presence non-hydrolyzable ATP (ATPγS) to enrich for the active form of Thorase. After several washes Thorase was cleaved from the GST-bound-beads with prescission protease to specifically elute protein complexes interacting with Thorase-ATPγS. We then separated the eluted complexes by fast protein liquid chromatography (FPLC), followed by unbiased identification of Thorase interactors via mass spectrometry (Fig. 1a). Among the putative Thorase-binding partners (Supplementary Table 1), mass spectrometry identified the mTOR kinase (Supplementary Fig. 1a, b). We decided to focus on this potential interaction between Thorase and mTOR because 1) similar to mTOR, Thorase promotes the survival of neurons^13^, and 2) mice lacking Thorase have abnormal phenotypes such as epileptic-like seizures^14,16,20,21^, similar to mice with aberrant mTORC1 activity and families with mutations in the mTOR pathway^22–26^.

To validate our mass spectrometry findings, we analyzed the fractionated protein complexes pulled down by recombinant Thorase via immunoblotting, and thus further confirmed that endogenous mTOR and recombinant Thorase co-exist in complexes of high molecular weight (Fig. 1b, FPLC fractions 2-3 in Supplementary Fig. 1c). Binding between Thorase and mTOR was direct, as revealed by our experiments performed in vitro, where purified recombinant mTOR and Thorase proteins alone assembled into high molecular weight complexes similar to those observed from brain pulldowns (Supplementary Fig. 1d, e). As expected, binding of Thorase and mTOR was strong in the presence of ATPγS, both when we pulled down endogenous mTOR (Fig. 1c–e) or in the reverse pull down of endogenous Thorase (Fig. 1f, g). In contrast, when we performed immunoprecipitation assays in the presence of hydrolyzable ATP or ADP, both pull down of mTOR by Thorase and pull down of Thorase by mTOR was less efficient (Fig. 1c–g), as it had previously been reported with other Thorase interactors^14,16^. Thus, ATP binding to Thorase is essential for Thorase-mTOR interaction.

Thorase preferentially binds to mTOR, rather than mTORC1-associated proteins Raptor, S6K or 4E-BP1 (Fig. 1f, g and Supplementary Fig. 1c). Concomitant to a strong binding between Thorase and mTOR, we detected a decrease in mTOR binding to the mTORC1 component Raptor, and mTORC1 substrates S6K and 4E-BP1 (Fig. 1c–f). A weaker interaction between Thorase and mTOR in the presence of ADP or hydrolyzable ATP correlated to an enhanced binding of mTOR to Raptor, 4E-BP1 and S6K (Fig. 1c–f). Moreover, in the absence of Thorase, Raptor, S6K and 4E-BP1 proteins co-immunoprecipitated with mTOR regardless of what nucleotide we added to the reaction (Fig. 1c–e, Supplementary Fig. 1f, g). The amount of mTORC1 proteins that dissociated from mTOR in the wild type samples (~80%) was about four-fold more than the Thorase knockout (KO) samples (~20%) consistent with the idea that Thorase is required for the disassembly of mTOR from Raptor, S6K and 4E-BP1 (Fig. 1e).

Providing evidence for the physiological relevance of this interaction, endogenous mTOR and Thorase co-localized in mouse embryonic fibroblasts (MEFs) and mouse brain sections (Supplementary Fig. 2a). Similar to what we found in pull down assays, in the absence of Thorase, we observed increased co-localization of mTOR with mTORC1-related proteins S6K and 4E-BP1, but not mTORC2-associated protein Rictor (Supplementary Fig. 2b, c). Overall, these results suggested that Thorase directly interacts with mTOR and that it may be involved in the disassembly of the mTORC1 (Fig. 1h).

### Thorase disassembles the mTORC1

**Fig. 2:**
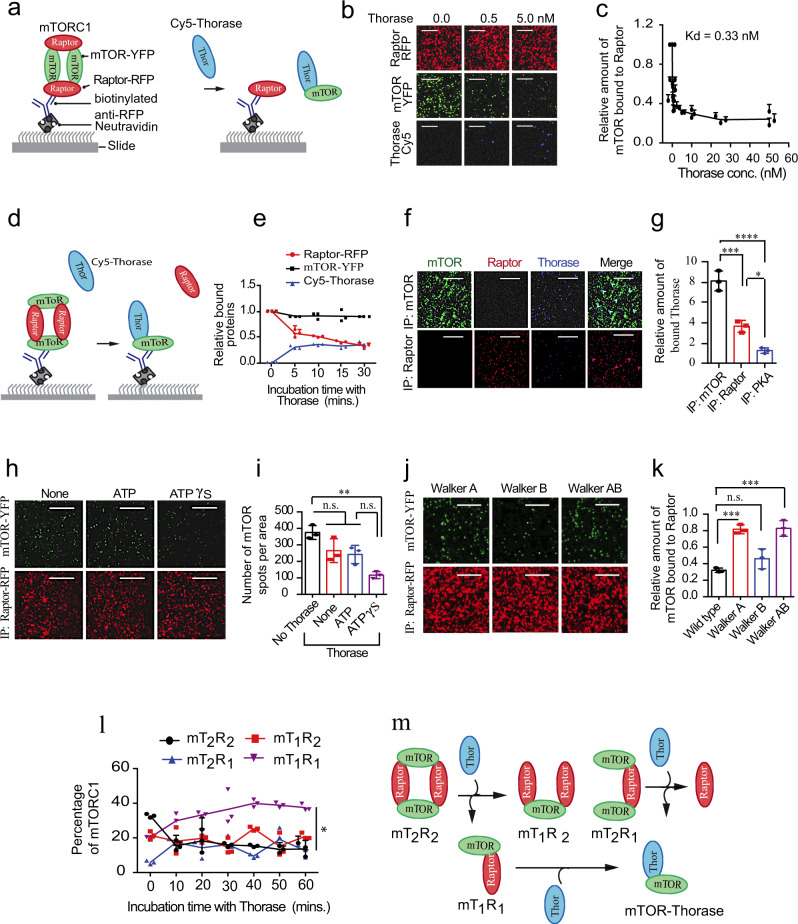
Thorase disassembles the mTOR complex 1 (mTORC1). **a** Schematic depiction of mTORC1 SiMPull in the presence of Thorase. Adapted from Jain et al.^28^. **b** Representative images from SiMPull shown in **a** with different concentrations of Thorase. Scale bars are 10 μm. **c** Graphical representation of the kinetics of Thorase disassembly of mTOR-Raptor complex with a Kd value of 0.33 nM. **d** Schematic diagram of mTOR-Pulldown via SiMPull showing Thorase binds to mTOR upon disassembly of Raptor from mTORC1. Adapted from Jain et al.^28^. **e** Graphical representation of Raptor (red) disassociation from mTOR and Thorase (blue) binding to mTOR over time (*n* = 3 independent SiMPull assays, Thorase-Cy5 values 5x). **f** Representative images of SiMPull binding analyses showing that Thorase has higher affinity for mTOR than Raptor. Scale bars are 10 μm. **g** Graphical representation of quantified intensities from images in **f**. **h** Representative images of Raptor-pulldown SiMPull analyses of mTOR-Raptor complex in the presence of Thorase and different nucleotides. Scale bars are 10 μm. **i** Quantification of number of mTOR spots per imaging area in **h** (*n* = 3 independent SiMPull assays). **j** Representative images of SiMPull analyses of mTOR-Raptor complex in the presence of different Thorase ATPase variants. Scale bars are 10 μm. **k** Quantification of images shown in **j** (*n* = 3 independent SiMPull assays). **l** Graphical representation showing the distribution of the different mTOR-Raptor species over time in the presence of Thorase (*n* = 3 independent SiMPull assays). **m** Schematic diagram of SiMPull showing Thorase disassembly of the mTOR-Raptor complex. Data in **g**, **i**, **k** are mean ± standard error of the mean [SEM] of three independent experiments, ***p < 0.001, *p < 0.05, n.s *p* > 0.05, analyzed with one-way ANOVA with Tukey’s post-hoc test (exact *p* values indicated in Data Source File).

To assess whether Thorase could disassemble the mTORC1, we used single-molecule pull-down (SiMPull), which combines traditional immunoprecipitation with single molecule fluorescence imaging, allowing for the study of the binding dynamics within protein complexes^27–29^. Previously, SiMPull has been successfully used to characterize the stoichiometry and assembly dynamics of the mTOR complexes^28^. We pulled down mTORC1 on slides from cell extracts by capturing the mTORC1-specific protein Raptor tagged with red fluorescent protein (RFP), and then imaged yellow fluorescent protein (YFP)-tagged mTOR and RFP-Raptor (Fig. 2a and Supplementary Fig. 3a). We used changes in the number of YFP and RFP spots on the slide as a proxy for the disassembly of mTORC1 complexes^27–29^. The addition of recombinant Thorase resulted in its dissociation from Raptor, as indicated by the decrease in the number of mTOR-YFP molecules on the slide (Fig. 2b and Supplementary Fig. 3b, c). The dissociation of mTOR from Raptor was dose-dependent and consistent with a high avidity binding of Thorase to mTOR, with a mid-point Thorase concentration of 0.33 nM (Fig. 2c). As a negative control for a potential lack of specificity of the actions of Thorase on mTOR, we observed no significant change in the number of RFP-Raptor spots after the addition of Thorase (Fig. 2b and Supplementary Fig. 3b, c), and neither did Thorase disassemble the PKA complex that was used as a control^27–29^ (Supplementary Fig. 3d–f). When we performed the capture of mTORC1 in an alternative approach via the pulldown of YFP-mTOR, and subsequently added Cy5-labeled Thorase onto the slide (Fig. 2d), the number of RFP-Raptor spots on the slide decreased while the number of Cy5-Thorase spots increased, suggesting that Thorase displaces Raptor by binding to mTOR (Fig. 2e and upper panels on Fig. 2f). To further support the specificity of Thorase-mTOR binding, rather than Thorase-Raptor interaction, we pulled down mTORC1 via capture of Raptor, or performed a control experiment with the PKA complex. In both cases, we detected a significantly smaller number of Cy5-Thorase molecules bound to Raptor and the PKA complex, compared to our earlier experiment with immobilized mTOR (lower panels on Fig. 2f, g).

Similar to what we had observed with traditional immunoprecipitation assays (Fig. 1 and Supplementary Fig. 1), mTOR disassembly from Raptor by Thorase is dependent on ATP binding, as addition of Thorase in the presence of the non-hydrolyzable ATP analog, ATPγS, promoted a sharper decrease in YFP-mTOR and RFP-Raptor spots, compared to Thorase addition in the absence of nucleotides, or in the presence of ATP (Fig. 2h, i and Supplementary Fig. 3g, h). Supporting the notion that ATP binding is needed the efficiency of disassembly of mTOR, as measured by disappearance of YFP-mTOR spots, decreased in the presence of Thorase mutants Walker A and Walker AB that are incapable of binding ATP, as compared to wild type Thorase or mutant Walker B, which can bind ATP but not hydrolyze it^14,16^ (Fig. 2j, k). Consistent with previous studies on mTORC1 assembly^27–29^, in the absence of Thorase the dimer forms of mTOR and Raptor were the dominant species. Following Thorase addition, monomeric mTOR and Raptor emerged as the dominant species (Supplementary Fig. 3i, j). Detailed stoichiometric analyses of the mTORC1 further confirms that the dimeric species, mT_2_R_2_, dominates in the absence of Thorase, whereas the monomeric species, mT_1_R_1_, dominates in the presence of Thorase (Fig. 2l and Supplementary Fig. 3k). This suggests that Thorase disassembles the mTORC1 by removing mTOR molecules one at a time (Fig. 2m). Collectively, these findings demonstrate that Thorase binds strongly and selectively to mTOR to mediate the disassembly of the mTORC1.

### Thorase negatively regulates mTORC1 signaling

**Fig. 3:**
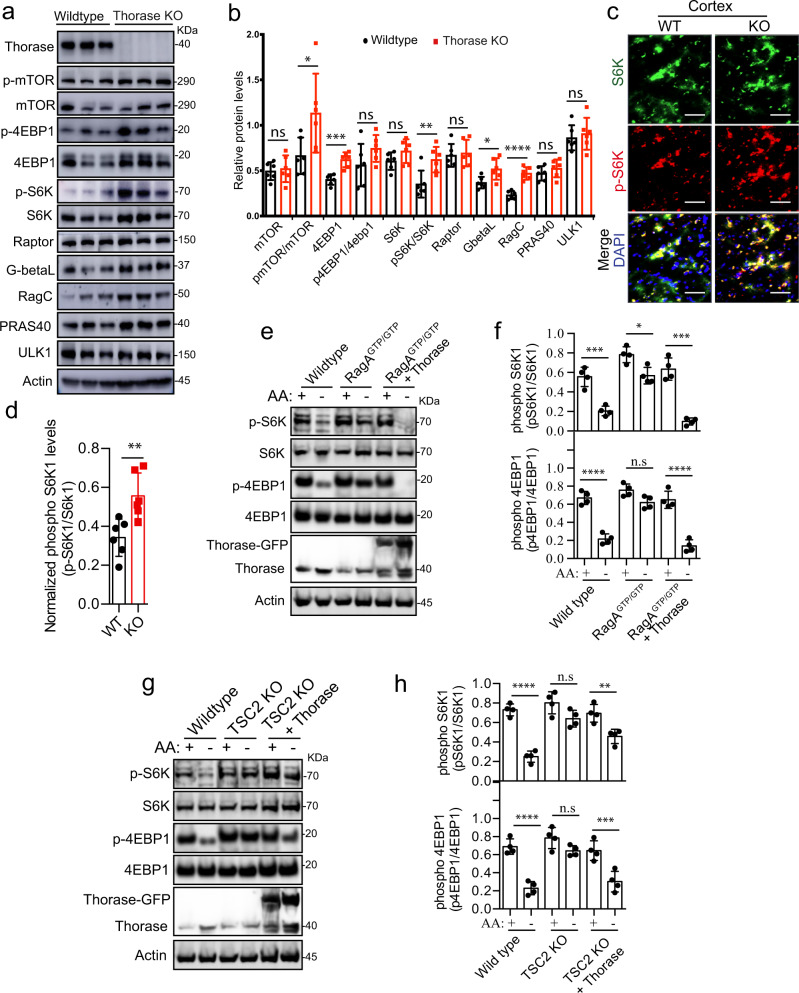
Thorase regulates the activity levels of the mTORC1 pathway. **a** Immunoblot images of mTORC1-associated proteins in wild type (WT) and Thorase knockout (KO) mouse brains. **b** Quantification of immunoblots in **a** (*n* = 6 biologically independent samples). **c**–**d** Representative immunostained images and quantification showing increased levels of phospho-S6K in KO mice cortex compared to WT (i = 6 biologically independent samples) Scale bars are 50 μm. **e** Immunoblot images showing Thorase over-expression normalizes elevated mTORC1 activity in RagA^GTP/GTP^ cells in the presence (+) or in the absence (−) of amino acids (AA). **f** Quantification of blots in **e** (*n* = 4 independent experiments). **g** Immunoblot images showing Thorase over-expression normalizes elevated mTORC1 activity in TSC2 knockout (KO) cells in the presence (+) or in the absence (−) of amino acids (AA). **h** Quantification of blots in **g** (*n* = 4 independent experiments). Data in **b**, **d**, **f** and **h** are mean ± standard error of the mean [SEM] of independent experiments, ***p < 0.001, **p < 0.01, *p < 0.05, n.s *p* > 0.05, **b** data from multiple unpaired t tests with Holm–Šídák test for multiple comparisons. **d** two-tailed unpaired *t*-test. **f**, **h** one-way ANOVA with Tukey’s post-hoc test (exact *p* values indicated in Data Source File).

Next, we assessed whether Thorase disassembly of mTORC1 had any consequences on steady state mTORC1 signaling. Total and phosphorylated levels of several mTORC1-related proteins were higher in brains of Thorase KO mice (Fig. 3a, b), suggesting elevated mTORC1 signaling occurs in the absence of Thorase. Phospho-S6K levels were also significantly elevated in Thorase KO mice as revealed by immunohistochemical staining of brain slices (Fig. 3c, d). We confirmed the same mTORC1 hyperactivity occurs in fibroblasts derived from Thorase KO mouse embryos, which presented levels of mTORC1 signaling twice as high in comparison to Thorase wild type fibroblasts (Supplementary Fig. 4a, b). Consistent with excessive mTORC1 activity in Thorase KO fibroblasts, the levels of newly synthesized proteins assessed by methionine-^35^S or puromycin incorporation were significantly higher (Supplementary Fig. 4c–e).

It is well established that mTORC1 signaling is regulated via the Tuberous Sclerosis Complex (TSC) and the Rag family of GTPases. Cells deficient of TSC2 (TSC2 KO) or with a constitutively active form of RagA GTPases (RagA^GTP/GTP^) are insensitive to mTORC1 activity regulation by amino acids, with cells displaying active mTORC1 signaling even under amino acid starvation conditions^1,7,30^. Since Thorase KO cells show hyperactive mTOR signaling similar to RagA^GTP/GTP^ and TSC2 KO cells, we assessed whether over-expressing Thorase in RagA^GTP/GTP^ and TSC2 KO cells could compensate for the aberrant mTORC1 signaling observed under amino acid starvation conditions. Over-expression of Thorase was able to counteract excessive mTORC1 signaling (S6K and 4E-BP1 phosphorylation) in these cells (Fig. 3e–h). Ectopic expression of Thorase both in RagA^GTP/GTP^ or TSC2 KO fibroblasts decreased the number of mTORC1 complexes, as measured by the number of mTOR/Raptor puncta (Supplementary Fig. 4f, g). Ectopic expression of Thorase in both hyperactive mTORC1 cell lines was also able to reduce mTOR recruitment by RagC (Supplementary Fig. 4h, i). These results taken together suggest that Thorase binding and subsequent disassembly of mTORC1 negatively regulates mTORC1 signaling.

### Thorase and mTOR interact at mitochondria-lysosome interfaces

**Fig. 4:**
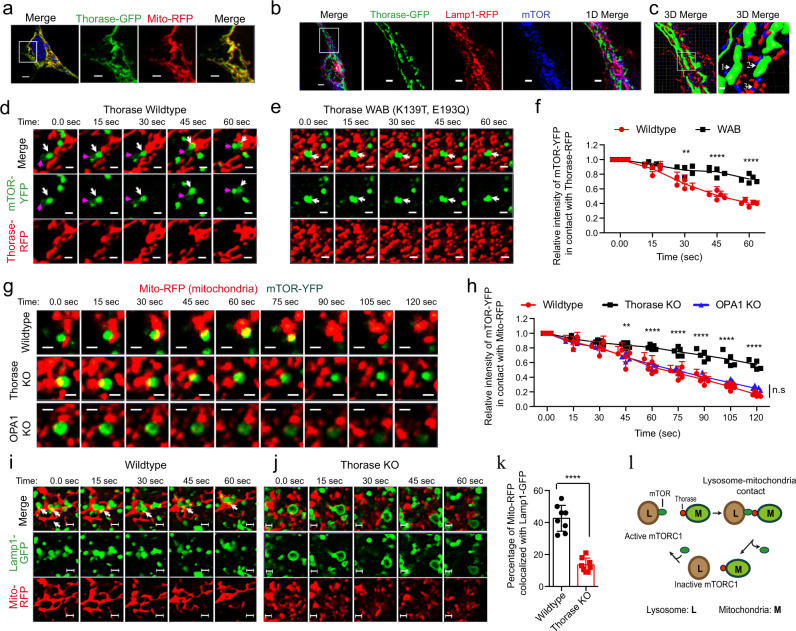
Thorase interacts with mTOR at the lysosome-mitochondria contacts. **a** Representative images of mouse embryonic fibroblasts (MEF) co-expressing Thorase-GFP (green) and mito-RFP (mitochondria, red). Scale bars are 5 μm (low), 2 μm (high) magnification. **b**, **c** Representative 2D and 3D Images of mouse embryonic fibroblasts (MEF) showing colocalization of Thorase and mTOR at the lysosome (Lamp1). Scale bars are 5 μm (low), 2 μm (higher) magnification. Different interactions, 1 (Thorase-mTOR), 2 (Thorase-mTOR-Lamp1) and 3 (mTOR-Lamp1) are indicated. **d** Representative images of time series of colocalization of wild type Thorase-RFP (red) with mTOR-YFP (green). The interactions of Thorase with mTOR vesicles (white arrow) results in diffusion or reduction of mTOR labels. Meanwhile mTOR labeling increases in vesicle (purple arrow) that is not in contact with Thorase-vesicles. Scale bars are 1 μm. **e** Representative images of time series of colocalization of Thorase-RFP WAB mutant (red) with mTOR-YFP (green). Scale bars are 1 μm. **f** Quantification of mTOR-YFP signal intensity in **d** and **e** (*n* = 5 independent experiments). **g** Representative images of time series of colocalization of mito-RFP (red) with mTOR-YFP (green) in wild type, Thorase KO and OPA1 KO MEFs. Scale bars are 1 μm. **h** Quantification of mTOR-YFP signal intensity in **g** (*n* = 5 independent experiments). **i**, **j** Representative images of time series of colocalization of Lamp1-GFP (green) with Mito-RFP (red) of wild type or Thorase KO MEFs. The lysosome-mitochondria contacts are indicated by arrows. Scale bars are 1 μm. **k** Quantification of number of lysosome-mitochondria contacts in (**i** and **j**) (*n* = 8 independent experiments). **l** Schematic diagram showing how Thorase at mitochondria (M) interacts with mTOR at the lysosome (L). Data in **f**, **h** and **k** are SEM of independent experiments, ***p < 0.001, **f** multiple unpaired t tests with Holm–Šídák test for multiple comparisons, **h** one-way ANOVA with Tukey’s post-hoc test, **k** two-tailed unpaired *t*-test (exact *p* values indicated in Data Source File).

The positive mTOR regulator Rheb is enriched at the lysosome, and recruitment of mTOR to this organelle by the Rag GTPases in response to amino acid availability is critical for the activation of the mTORC1 pathway^7,12,31^. Therefore, we sought to evaluate whether the inhibitory actions of Thorase over mTORC1 occurred at lysosomes. We and other groups have described the predominant localization of Thorase to the outer membrane of mitochondria, where it serves a role in organelle integrity^17,18^. We have also previously shown that Thorase-GFP, like the endogenous protein, is active^16^, and localized in mitochondria when ectopically expressed in MEFs (Fig. 4a). We found that in MEFs Thorase-GFP localized near lysosome-bound mTOR (Fig. 4b). 3D rendering of this co-localization suggests that Thorase directly interacts with mTOR on the surface of lysosomes without directly binding to the lysosome (Fig. 4c). Live imaging with YFP-tagged mTOR (mTOR-YFP) and Thorase-RFP revealed that mTOR-YFP puncta diffused after interacting with Thorase-RFP at the mitochondria (Fig. 4d, arrows, Supplementary movie 1). In the presence of Thorase ATPase deficient mutant, WAB (K139T, E193Q)^14^ we observed less mTOR-YFP puncta diffusion (Fig. 4e arrows, Fig. 4f). Further evaluation of mTOR-YFP puncta diffusion in wild type and Thorase KO MEFs expressing mTOR-YFP with Mito-RFP (mitochondria) suggests that the rate of mTOR-YFP puncta diffusion is significantly decreased in Thorase KO compared to wild type cells (Fig. 4g, h, Supplementary movie 2). To rule out that the decreased rate of mTOR puncta diffusion in Thorase KO cells could be due to the presence of fragmented mitochondria in these cells^17^, we examined the rate of mTOR puncta diffusion in cells lacking the mitochondria fusion protein OPA1 (OPA1 KO), which also exhibit fragmented mitochondria^32,33^. Unlike Thorase KO, OPA1 KO cells exhibit a rate of mTOR puncta diffusion similar to wild type (Fig. 4g, h), demonstrating that the decreased rate of mTOR puncta diffusion is specific to the loss of Thorase. Taken together, our observations indicate that mTOR dissociates from lysosomes upon contact with mitochondria bound Thorase and that Thorase ATPase activity is important for the dissociation of mTOR from the lysosome.

Mitochondria-bound Thorase formed discrete contacts with lysosome marker Lamp1 in our live imaging experiments. This was not an artifact of protein overexpression, as only Thorase-GFP but not GFP alone co-localized with Lamp1-RFP (Supplementary Fig. 5a, b and Supplementary movie 3). Mitochondria-lysosome contacts have recently been characterized beyond the context of mitophagy, and these contacts are believed to be important to regulate organelle dynamics and mTORC1 signaling^34–37^. We thus wondered whether the absence of Thorase would have any impact on the mitochondria-lysosome contacts found in the cell. Co-localization studies in wild type and Thorase KO MEFs revealed that the number of mitochondria-lysosome contacts were reduced by half in the absence of Thorase (Fig. 4i–k), suggesting Thorase may mediate inter-organelle tethering. Overall, these observations suggest that Thorase plays an important role in regulating mTORC1 signaling at the mitochondria-lysosome interfaces (Fig. 4l).

### Thorase disassembly of mTORC1 is sensitive to amino acid availability

**Fig. 5:**
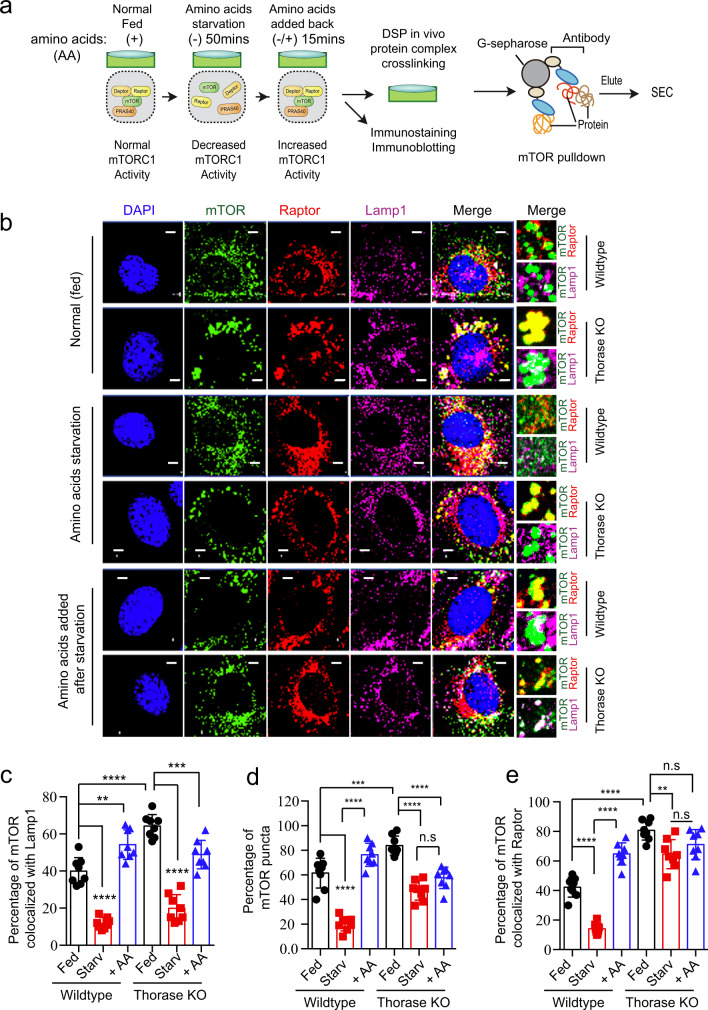
Thorase modulates mTOR interaction with Raptor for activation at the lysosomes by amino acids. **a** A schematic diagram depicting the protocol for analysis of Thorase-mTOR-Raptor complexes under different nutrient status in MEFs. **b** Representative images of wild type and Thorase KO MEFs under different nutrient status showing mTOR-Raptor and mTOR-Lamp1 co-localization. Scale bars are 5 μm. **c** Quantification of mTOR-Lamp1 colocalization of images in **b** (*n* = 8 independent experiments). **d** Quantification of mTOR puncta of images in **b** (*n* = 8 independent experiments). **e** Quantification of mTOR-Raptor colocalization of images in **b** (*n* = 8 independent experiments). Data are mean ± standard error of the mean [SEM] of experiments performed, ***p < 0.001, **p < 0.01, *p < 0.05, n.s *p* > 0.05, analyzed with two-way ANOVA with Tukey’s post-hoc test (exact *p* values indicated in Data Source File).

Amino acid starvation inhibits mTORC1 activity, and mTORC1 can be re-activated at the lysosome when nutrients are available again^31,38^. Thus, Thorase disassembly of mTORC1 and consequent regulation of its activity might be most relevant upon amino acid starvation. As such, we sought to determine whether the binding between Thorase and mTOR and subsequent dissociation from mTORC1 components was sensitive to amino acid availability (Fig. 5a). In fed wild type MEFs, about 40% of mTOR was co-localized with LAMP1-positive lysosome puncta (first row of Fig. 5b, c) and 60% mTOR puncta (Fig. 5d). During amino acid starvation in wild type cells, mTOR signal diffused throughout the cytosol, and only about 20% of mTOR signal remained punctate (5b–d). Within 10 min of re-feeding with amino acids, mTOR re-localized to LAMP1 puncta, with around 60% of mTOR signal co-localizing with LAMP1 and 80% mTOR signal becoming punctate (Fig. 5b–d) as previously suggested^7,39^. Thorase KO MEFs had around 1.5 times more LAMP1-positive puncta containing mTOR, although mTOR diffused to the cytosol as efficiently during starvation as in wild type MEFs (Fig. 5b, c). In wild type MEFs, mTOR levels at the lysosome 10 min after re-feeding with amino acids surpassed the lysosomal mTOR levels at steady state (Fig. 5c). In contrast, in Thorase KO MEFs, re-feeding with amino acids did not result in lysosomal mTOR levels above baseline, but rather mTOR was found at the lysosome in levels below re-fed wild type MEFs, around 55% (Fig. 5c).

**Fig. 6:**
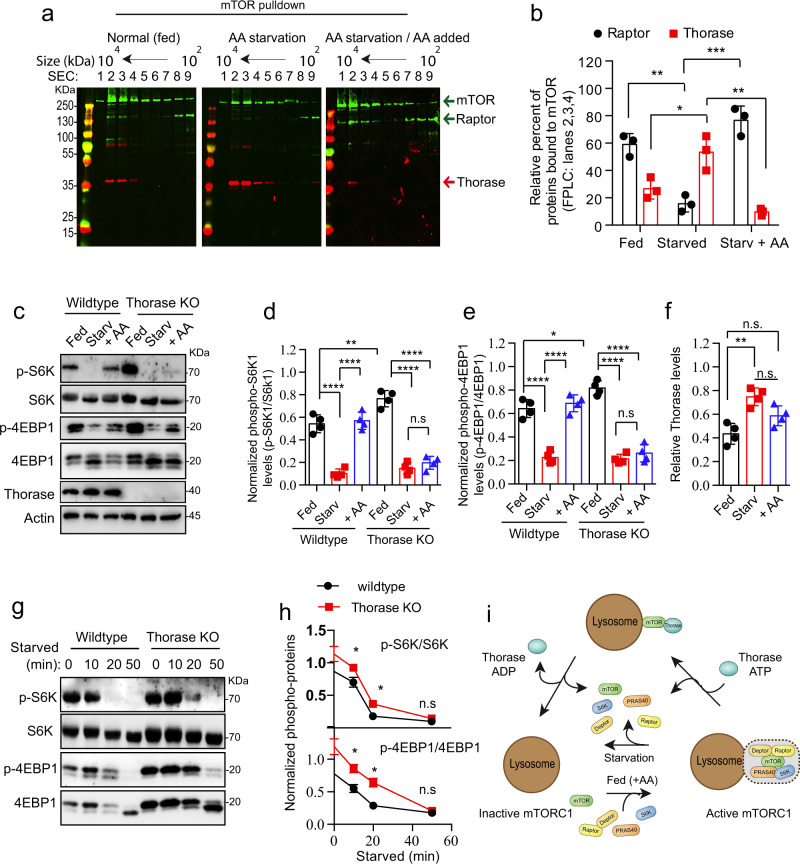
Amino acids stimulation of mTORC1 activation delay in the absence of Thorase. **a** Representative immunoblot images of mTOR IP (pulldown) protein complexes from WT MEFs under different nutrient status and protein complexes separated by SEC. **b** Quantification of blots in **a** (*n* = 3 independent pull downs). **c** Representative immunoblots of WT and KO MEFs showing mTORC1 activation (phosphorylation) status during amino acids (AA) starvation (Starv) and/or amino acid supplementation (+ AA). **d**–**f** Quantification of relative levels of phospho-S6K (**d**), phospho-4E-BP1 (**e**), and Thorase (**f**) in **c** (*n* = 4 independent experiments). **g**, **h** Representative immunoblots of WT and KO MEFs showing mTORC1 proteins activation (phosphorylation) status during amino acid starvation at different times and quantification of relative levels of phospho-S6K and phospho-4E-BP1 (*n* = 3 independent experiments). **i** A schematic diagram depicting the disassembling of the mTORC1 complex under different nutrient status in the presence of Thorase. Data in **b**, **d**, **e**, **f**, and **h** are mean ± standard error of the mean [SEM] of experiments performed, ***p < 0.001, **p < 0.01, *p < 0.05, n.s *p* > 0.05. **b**, **f** one-way ANOVA with Tukey’s post-hoc test, **d**, **e** two-way ANOVA with Tukey’s post-hoc test, **h** two-tailed unpaired *t* test (exact *p* values indicated in Data Source File).

We next assessed mTOR and Raptor binding as a proxy of mTORC1 complex integrity at different feeding states. In wild type MEF, Raptor/mTOR co-localization decreased upon starvation, and paralleling mTOR-LAMP1 co-localization, it was significantly higher than baseline 10 min after cells were re-fed with amino acids (Fig. 5b, e). In Thorase KO MEFs, steady state levels of Raptor/mTOR co-localization doubled in comparison to wild type cells but, unlike in wild type MEFs, the stability of these complexes was not largely altered by either amino acid starvation or re-feeding (Fig. 5e). We obtained similar results in our immunoprecipitation of mTOR and size exclusive chromatograph (SEC) experiments (Fig. 6a, b). Starving cells from amino acids decreased the amount of Raptor that was pulled down along with mTOR, and mTOR/Raptor binding increased when re-feeding cells with amino acids (Fig. 6b). In contrast, binding between Thorase and mTOR was strongest during starvation and interaction levels decreased sharply upon amino acid re-feeding.

In wild type MEFs fed with amino acids mTOR phosphorylates S6K and 4E-BP1, when amino acids are removed from media this phosphorylation decreases, and within 10 min of re-feeding cells with amino acids phosphorylation returns to steady state levels^7^. As expected, the relative interaction of mTOR with Raptor and RagC was higher in steady state (fed) conditions and after amino acid re-addition, while it was lower in the absence of amino acids (Supplementary Fig. 5c, d). mTOR/Thorase interaction followed the opposite pattern; during amino acid starvation, we detected a significant increase in Thorase levels both in whole cell lysates and mTOR pulldowns (Fig. 6a, b, f and Supplementary Fig 5c, d). Overall, these data support the notion that Thorase exerts its actions on mTORC1 during amino acid depletion, when mTORC1 signaling needs to be inhibited.

Consistent with our observations that mTOR localization at the lysosome decreases upon starvation in Thorase KO MEFs, we observed a decrease in the phosphorylation levels of S6K, and 4E-BP1, and a shift in 4E-BP1 isoform migration (Fig. 6c). However, unlike in wild type MEFs, we failed to detect an increase in phospho-S6K levels and the higher molecular weight 4E-BP1 isoform within 10 min of re-feeding Thorase KO MEFs (Fig. 6c–e), suggesting a delay in re-activation of mTORC1 in Thorase KO cells. A closer look at the dynamics of starvation revealed that Thorase KO MEF starvation was also delayed compared to wild type cells (Fig. 6g, h and Supplementary Fig. 5e–g). Although we had previously observed effective removal of mTOR from lysosomes upon 50 min of amino acid starvation, live imaging experiments of Thorase KO MEFs revealed there was a delay in removing mTOR from the lysosome in the first few minutes of amino acid starvation (Supplementary Fig. 5h–j). Overall, these results suggest that Thorase is necessary for proper mTORC1 disassembly during starvation, and that lack of Thorase results in untuned mTORC1 regulation by amino acids (Fig. 6i).

To rule out the possibility that Thorase may regulate the recruitment of mTOR to the lysosome via altering Rag protein levels or directly influencing mTOR-Rag protein binding, we first evaluated Rag protein levels in both Thorase KO brains and fibroblasts. RagC levels were significantly higher in both Thorase KO brains and fibroblasts compared to control, while RagA levels were significantly higher only in Thorase KO fibroblasts (Supplementary Fig. 6a–d). We detected no significant differences in terms of RagD protein levels (Supplementary Fig. 6a–d). However, we did not observe significant changes in RagC localization in wild type versus Thorase KO cells (Supplementary Fig. 6e, g). mTOR was able to localize away from RagC under amino acid starvation to levels comparable to wild type cells, and the percentage mTOR/RagC co-localization 15 min after amino acid re-addition was not significantly different to steady state (fed) conditions (Supplementary Fig. 6e, f). Although steady state mTOR/RagC puncta were more abundant in Thorase KO cells, mTOR/RagC binding dropped during starvation, and increased after amino acid re-addition, as expected in wild type cells (Supplementary Figs. 5c, d, 6e, f).

Given RagC levels were significantly elevated, we next evaluated whether an increased mTOR/RagC association could lead to the elevated mTORC1 signaling in the absence of Thorase. Our immunofluorescence detection of mTOR/RagC/Lamp1 suggest that in steady state (fed) conditions, co-localization of mTOR with RagC is significantly higher in Thorase KO fibroblasts than in wild type counterparts, consistent with our findings that steady state localization of mTOR at the lysosome (using Lamp1 as proxy) was higher in the absence of Thorase (Fig. 5c). However, mTOR was able to localize away from RagC under amino acid starvation to levels comparable to wild type cells, and the percentage mTOR/RagC co-localization 15 min after amino acid re-addition was not significantly different to steady state (fed) conditions. RagC localization itself was also not altered in Thorase KO cells, regardless of their nutrient status. Overall, these findings suggest Thorase does not alter the interaction between mTOR and Rag proteins, nor Rag protein localization.

mTORC1 activation occurs upon coincidental availability of amino acids and growth factors (reviewed in ref. 8). To assess whether availability of growth factors is also important for the effects of Thorase on mTORC1, we used a serum starvation paradigm followed by insulin stimulation^40,41^ (Supplementary Fig. 6h–k). Thorase KO embryonic fibroblasts showed elevated phosphorylated levels of phospho-S6K, phospho-S6 and phospho-4E-BP1 when fed with media containing both growth factors and amino acids (fed). The activity of phospho-S6K and phospho-S6 decreased upon serum starvation, just as much as in their wild type counterparts (Supplementary Fig. 6i, j). 4E-BP1 phosphorylation levels were not affected by the lack of serum, regardless of the genotype (Supplementary Fig. 6k). 15 min of insulin stimulation induced the phosphorylation of all mTORC1 effectors, and their levels in Thorase KO cells were equivalent to that in wild type cells. As opposed to the differences observed at steady state conditions (fed), we did not observe significant differences in mTORC1 activity between wild type and KO cells 15 min after insulin stimulation. Our results suggest that these differences arise from the long-term failure of Thorase to regulate the mTORC1 pathway. These observations upon serum starvation are in contrast to our results from amino acid depletion and replenishment, where Thorase KO cells failed to promptly stimulate the mTORC1 pathway like wild type counterparts. These differing outcomes indicate that mTORC1 regulation by Thorase is strongly impacted by amino acid availability over growth factor availability.

### Thorase N-terminal end is key for mTORC1 disassembly and inhibition

We next sought to gain more insight into the mechanics of Thorase inhibition of mTORC1 at a molecular level. To determine what Thorase domains are responsible for its interaction with mTOR, we generated N-terminal (N40, N50, N60, N70, N90, N100) and C-terminal (C10 and C20) truncated variants (Supplementary Fig. 7a). Both pull downs with GST-Thorase and mTOR suggest that deletion of the residues 70-90 on the N-terminal end of Thorase inhibits its binding to mTOR (Supplementary Fig. 7b–e). Similarly, SiMPull analyses confirm that the N-terminus of Thorase is critical for binding to mTOR since mutants lacking the first hundred amino acid residues of Thorase disassemble the mTOR-Raptor complex less efficiently (Supplementary Fig. 7f, g). Thorase mutants N40 and C20, which have the first 40 and last 20 amino acid residues deleted, respectively, can effectively localize to the lysosome, whereas the N100 mutant lacking the first 100 amino acid residues cannot (Supplementary Fig. 7h). To assess whether mTORC1 phenotypes are direct and dependent on mTOR-Thorase binding, we quantified mTOR/Lamp1 and mTOR/Raptor co-localization in Thorase KO cells ectopically expressing WT Thorase or N100 Thorase truncation, which does not bind mTOR (Supplementary Fig. 7i). In Thorase KO fibroblasts ectopically expressing full length (WT) Thorase, mTOR/Raptor co-localization was significantly lower in steady state (fed) conditions compared to untransformed Thorase KO fibroblasts (Supplementary Fig. 7i, j). The percentage mTOR/Raptor interaction significantly decreased upon removal of amino acids, and mTOR/Raptor interaction was significantly increased after amino acid re-addition when compared with WT Thorase transformed fibroblasts in fed conditions (Supplementary Fig. 7i, j). In contrast, Thorase KO fibroblasts transformed with the N100 truncation showed a mTOR/Raptor interaction that remained high regardless of the nutrition status, at levels comparable to mTOR/Raptor interaction in untransformed Thorase KO cells (Supplementary Fig. 7i, j). Similarly, Thorase mutants N40 and C20, which can bind mTOR, can reverse the increased levels of mTORC1 signaling observed in Thorase KO fibroblasts to a similar extent as wild type Thorase, while N100 mutant fails to do so (Supplementary Fig. 7k, l). These data indicate that Thorase N-terminal residues Gln^70^-Lys^90^ are important for interaction with and disassembly of the mTORC1, and that lack of Thorase-mTOR binding is directly responsible for the phenotypes observed in Thorase KOs.

### Rapamycin neutralizes the mTORC1 hyperactivity due to lack of Thorase

**Fig. 7:**
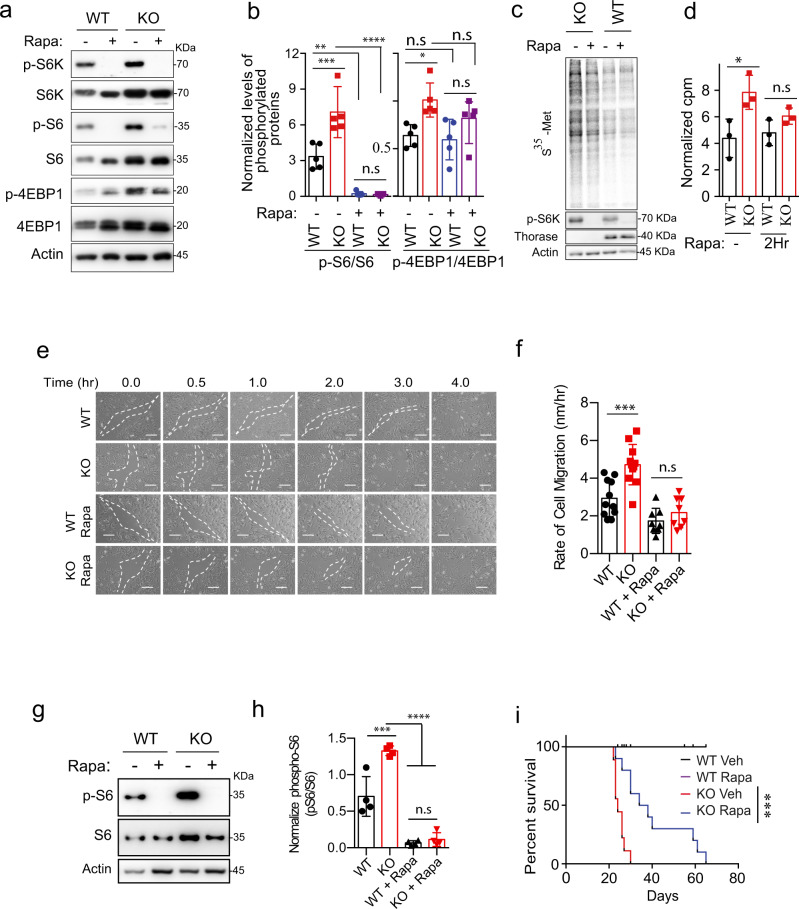
Rapamycin ameliorates phenotypes arising from Thorase dependent abnormal mTORC1 activation. **a** Immunoblot images showing rapamycin (Rapa) treatment normalizes elevated mTORC1 activity in Thorase KO cells. **b** Quantification of blots in **a** (*n* = 5 independent experiments). **c** Representative images showing rapamycin (Rapa) inhibits increased protein synthesis in Thorase KO cells. **d** Quantification of blots in **c** (*n* = 3 independent experiments). **e** Representative MEF migration images showing Rapa treatment normalizes increased cell growth and migration in Thorase KO MEFs. Broken white lines show areas without cells (scratched areas). Scale bars are 100 μm. **f** Quantification of images in **e** (*n* = 11 control and 8 rapamycin treatments). **g** Immunoblot images showing Rapa treatment normalizes elevated mTORC1 activity in KO mice cortex. **h** Quantification of blots in **g** (*n* = 4 biologically independent samples). **i** Survival curve of mice treated with rapamycin or vehicle (Veh) (Log Rank Mantel–Cox test, WT Veh *n* = 12 animals; WT Rapa *n* = 9 animals; KO Veh *n* = 9 animals; KO Rapa *n* = 10 animals; median survival KO Veh 24 days, KO Rapa 36 days). Data in **b**, **d**, **f**, **h** are mean ± standard error of the mean [SEM] of experiments performed, ****p < 0.0001, ***p < 0.001, **p < 0.01, *p < 0.05, n.s *p* > 0.05. **b**, **d**, **f**, and **h** two-way ANOVA with Tukey’s post-hoc test and **i** Log Rank Mantel–Cox test (exact *p* values indicated in Data Source File).

Our data demonstrate that Thorase binding to mTOR and subsequent disassembly of the mTORC1 is important to negatively regulate mTORC1 signaling. Therefore, we tested whether the mTOR inhibitor, rapamycin, could alleviate the mTORC1 hyperactivity observed when Thorase is lacking. Indeed, the levels of phosphorylated S6 in fibroblasts derived from either Thorase KO mice or wild type littermates decreased significantly after the treatment with rapamycin as compared to vehicle-treated counterparts (Fig. 7a). Although phosphorylated S6 and 4E-BP1 levels were higher in Thorase KO MEFs treated with vehicle, in rapamycin-treated MEFs the phospho-S6 levels were equally as low with either genotype (Fig. 7a, b). In contrast and as expected^42,43^, the levels of the rapamycin resistant T37/46 site in 4E-BP1 did not significantly change in either wild type or Thorase KO fibroblasts treated with rapamycin (Fig. 7a, b). Consequent to the rapamycin treatment, the synthesis of new proteins in these fibroblasts also decreased, as measured by incorporation of radiolabeled methionine (Fig. 7c, d).

Increased mTORC1 signaling causes abnormal cell growth and migration^44,45^. We therefore determined whether the absence of Thorase would have any effect on mTOR mediated cell migration. Our live imaging studies suggest that Thorase KO MEFs have increased cell growth and migration rates compare to wild type (Fig. 7e, f and Supplementary movie 4). Treating Thorase KO MEFs with rapamycin was able to restore cell migration rates to wild type levels as well. Together, these results demonstrate that the excess mTORC1 activity resulting from the lack of Thorase can be partially compensated with the mTOR kinase inhibitor rapamycin.

Thorase KO mice have a very short lifespan, with most mice dying two to three weeks after birth from epileptic-like seizures^14^. To test whether mTORC1 hyperactivity is accountable for some of the pathology underlying the sudden death of Thorase KO mice, we treated mice with rapamycin. Similar to what we observed in MEFs, intraperitoneal administration of rapamycin in both wild type and Thorase KO mice penetrated and effectively silenced mTORC1 activity in the brain (Fig. 7g, h). Decreasing mTOR activity with rapamycin lengthened the lifespan of mice lacking Thorase, with a significant increase in their median survival compared to vehicle treated Thorase KO mice (Fig. 7i). Some mice almost tripled their life span surviving for over 60 days postnatally, compared to vehicle-treated KO littermates that lived a median of 24 days. Finally, we video-recorded these mice around the clock to capture their death. While vehicle-treated mice died from severe seizures (Supplementary movie 5), mice treated with rapamycin died from what it seemed a progressive paralysis of the hindlimbs and eventual inability to feed themselves. Together, these results indicate that, in mice lacking Thorase, the increase in mTOR activity accounts for their lethality to a great extent.

## Discussion

Cells must metabolically adapt to the availability of nutrients, growth factors and energy, and to cellular stress. The mTOR kinase, as part of the mTORC1, achieves that by balancing the activation of energy-producing and -consuming metabolic activities in response to changes in both the extracellular and the intracellular environment. mTORC1 is recruited to the lysosomal surface by the heterodimeric Rag GTPases to enable its allosteric activation by the lysosome bound GTPase Rheb, upon coincident detection of nutrients and growth factors^31,34,46,47^. In turn, several proteins and protein complexes control the nucleotide status of Rags and Rheb GTPases to ensure timely and fine-tuned mTORC1 signaling^24,48^. Raptor interaction with active Rags is pivotal for mTORC1 recruitment to the lysosome^9^, but studies investigating whether mTOR/Raptor association itself is regulated and sensitive to the presence of growth factors and nutrients are lacking.

In the present study we have shown that AAA + ATPase Thorase binds the mTOR kinase directly both via traditional immunoprecipitation from mouse brains and in vitro via single molecule pulldowns (SiMPull), thereby preventing its binding to other mTORC1 components and breaking apart mTOR-Raptor complexes. We have demonstrated that Thorase disassembly of the mTORC1 is pivotal to negatively regulating its downstream signaling, as we detect excessive mTORC1 activity, including excess bulk protein synthesis, in cells and mice lacking Thorase. Ectopic expression of Thorase in two independent cell lines characterized by excessive mTORC1 recruitment and activation at the lysosome (RagA^GTP/GTP^ and TSC2 KO fibroblasts) was able to partially reduce the number of mTOR/Raptor complexes and the amount of mTOR recruited by the RagC, thus restoring mTORC1 signaling, further supporting the role of Thorase as negative regulator of the pathway. In addition, we show that the excess mTORC1 activity in Thorase KO cells and mouse brains can be counteracted with the allosteric mTOR inhibitor, rapamycin.

**Fig. 8:**
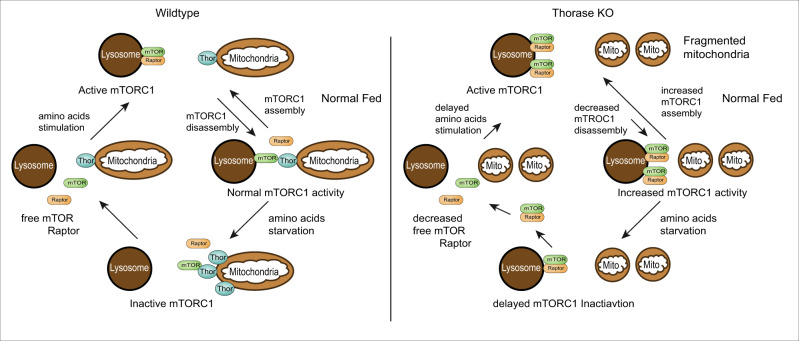
A schematic diagram of the proposed mechanism of how Thorase regulates mTORC1 activity. Under normal fed condition in wild type cells, mTORC1 is activated at the lysosome. Our data suggest that mTORC1 is disassembled upon lysosome contact with mitochondria where Thorase disassociate mTOR from the mTORC1 complex. During amino acid starvation, increased levels of Thorase at mitochondria results in the disassembly of mTORC1 and disassociation of mTORC1 from lysosome leading to free mTOR and other mTORC1 components (e.g., Raptor). Upon amino acids stimulation, the free mTOR and mTORC1 components rapidly re-assemble to form an active mTORC1 at the lysosome. In the absence of Thorase (Thorase KO), under normal fed condition mTORC1 disassembly is decreased resulting in more active mTORC1 at the lysosome and excessive mTORC1 signaling. During starvation, there is delayed disassociation of mTORC1 from lysosome. Our data further suggest that the mTORC1 disassociated from lysosome are still intact with fewer free mTOR and mTOR components. Amino acid stimulation of mTORC1 activity is delayed due to reduced or fewer free mTOR and mTORC1 components available in the KO cells.

AAA + ATPases are known to utilize the energy from ATP hydrolysis to assemble and disassemble protein complexes. To that extent, we previously found that Thorase disassembled GluA2/GRIP1 complexes in the presence of ATP^14,16^. In this study, both from traditional immunoprecipitation assays and SiMPull, which indicates that ATP binding, rather than hydrolysis, is required for the interaction between Thorase and mTOR, and that this interaction disassembles the mTORC1; similarly, our data with the Walker mutants seems to indicate that ATP binding, not hydrolysis is important to pull off mTOR, as the walker B mutant which binds ATP, but does not hydrolyze it pulls away mTOR to the same degree than the wild type protein (Fig. 2j, k). It is widely believed that ATP hydrolysis is not used for the power stroke in some motors but as a way of resetting the system for additional activities by the enzyme ATPases^49–51^. Accordingly, it is possible that Thorase only needs ATP binding for the action of mTORC1 disassembly but requires its hydrolysis for catalytic function, i.e., performing the action on more than one mTORC1 as our SiMPull experiments suggest (Fig. 2h, i). In the presence of ATP, the binding of ATP to Thorase allows Thorase to bind to mTOR in a manner that causes the disassociation of the mTORC1 proteins from mTOR. Upon the hydrolysis of ATP to ADP Thorase disassociates from mTORC1. Thorase disassociation from mTOR is critical for reactivating/assembling the mTORC1. After Thorase disassembles the mTORC1, the transient Thorase-mTOR complex disassociates upon ATP hydrolysis to allow reactivation of the mTORC1. Thus, Thorase has different mechanisms of action depending on what substrate it binds. Future studies are required to investigate these differences at the mechanistic level. A schematic diagram of our proposed mechanism of how Thorase regulates mTORC1 activity is provide in Fig. 8.

Our immunofluorescent and live imaging experiments indicate that Thorase interacts with mTOR at mitochondria-lysosome interfaces, where Thorase is predominantly compartmentalized in the cell and mTOR is shuttled for activation, respectively (Fig. 4). In the absence of Thorase, mitochondria appear fragmented, and the number of lysosome-mitochondrial contacts is decreased, suggesting that Thorase plays an important role in the establishment of such contacts (Fig. 4i–k). The importance of cell signaling at mitochondria-lysosome interfaces has been recently described^34–37^, implying that loss of Thorase could have major consequences in the signaling between these organelles.

We also explored whether Thorase regulation of mTORC1 was sensitive to amino acids (Figs. 5, 6 and Supplementary Fig. 5). Thorase-mTOR interactions and consequent mTORC1 disassembly peaked upon amino acid removal, at the time when mTORC1 signaling needs to be silenced (Fig. 6b). In Thorase-deficient cells, mTOR-Raptor complexes were numerous and were not pulled apart during starvation (Fig. 5e). This is likely why we observed a delay in the downregulation of mTORC1 upon removal of amino acids (Fig. 6g, Supplementary Fig. 5e–g), as well as the abnormally high steady state levels of mTORC1 signaling in cells and mice lacking Thorase (Fig. 3a–d and Supplementary Fig. 4a, b). Although delayed, mTORC1 signaling eventually fades upon sustained starvation (Fig. 6g and Supplementary 5e–g), suggesting that additional mechanisms exist to ensure termination of mTORC1 activity. For instance, it was shown recently that in nutrient-limited conditions Rap1-GTPases concentrate lysosomes to the perinuclear region and reduce overall lysosome abundance, thereby decreasing the surface available for mTORC1 activation^52^.

One possible way to explain excess mTORC1 signaling in Thorase KOs could be a direct regulation by Thorase of Rag protein levels, their localization, or ability to recruit and release mTORC1. We observed increased RagC levels in Thorase KO brains, and elevated RagC and RagA levels in Thorase KO fibroblasts (Supplementary Fig. 6a–d), consistent with our observations with other mTORC1 pathway proteins (Fig. 3a, b and Supplementary 4a, b). However, we did not observe significant changes in RagC localization in wild type versus Thorase KO cells (Supplementary Fig. 6e, g). mTOR was able to localize away from RagC under amino acid starvation to levels comparable to wild type cells, and the percentage mTOR/RagC co-localization 15 min after amino acid re-addition was not significantly different to steady state (fed) conditions (Supplementary Fig. 6e, f). Although steady state mTOR/RagC puncta were more abundant in Thorase KO cells, mTOR/RagC binding dropped during starvation, and increased after amino acid re-addition, as expected in wild type cells (Supplementary Figs. 5c, d, 6e, f). Overall, these findings suggest Thorase does not alter the interaction between mTOR and Rag proteins, nor Rag protein localization. Thus, in the absence of Thorase, mTOR could still be removed from the lysosome, indicating that shuttling mTORC1 back to the cytosol and breakdown of the complex are separable events. A recent study showed that Rag GTPases cycle spatially between lysosomes and the cytosol, thus likely minimizing the amount of time mTORC1 spends interacting with Rheb at the lysosomal surface^7,46,47^.

mTOR is a central signaling hub in cells and, as such, a wide range of human diseases have been attributed to aberrant function of this pathway, including metabolic and neurological diseases^2,22,45^. As one of the most energy-demanding organs in the body, the brain is particularly susceptible to metabolic dysregulation. Our study supports the idea that Thorase loss-of-function leads to chronic mTORC1 upregulation, with severe neurological consequences. Mice lacking Thorase die perinatally from what seem like epileptic-like seizures and administration of the mTOR inhibitor, rapamycin, significantly extended the lifespan of Thorase KO mice. This lifespan prolongation of Thorase KO mice treated with rapamycin is consistent with the idea that mTOR plays a role in their profound phenotype. Previously, we had extended the lifespan of Thorase KO mice from 20 to 43 days (*n* = 5 control, *n* = 8 treatment) by treating them with the AMPAR antagonist Perampanel, which was consistent with the role of Thorase in disassembling AMPAR complexes in neurons^20^. This preclinical study was used to modify the clinical treatment of patients with loss-of-function Thorase mutations^20^. 6 mg/kg rapamycin IP injection every other day prolonged median survival of Thorase KO mice from 24 days to 36 days (*n* = 9 control, *n* = 10 rapamycin, with Mantel–Cox Log-rank test showing the survival curves were significantly different at significance level below 0.0001) (Fig. 7i). A third of our rapamycin-treated Thorase KO mice lived more than 50 days, thus they more than doubled the median lifespan of vehicle-treated Thorase KOs. Therefore, the contribution of mTOR signaling to Thorase KO phenotypes are as important as the contribution to AMPAR-mediated hyperexcitability. We also do not rule out the possibility that both pathways may synergistically explain the severe pathology of Thorase KO mice.

Our findings on the role of Thorase in negatively regulating the mTORC1 pathway are likely to be relevant to patients harboring Thorase mutations^16,17,20,21^. Neurodevelopmental disorders associated with hyperactive mTOR signaling, also known as mTORopathies, are caused by loss-of-function mutations in negative regulators of mTORC1, and usually manifest with symptoms that include epileptic seizures and macrocephaly. A subset of patients that harbor Thorase mutations present symptoms that overlap with mTORopathies, and future studies will aim to elucidate whether excess mTOR activity underlie their pathology.

Studies of the yeast Thorase homologue, MSP1 indicate that Thorase has an evolutionarily conserved function that extends beyond the nervous system^17,18,53–55^. Consistent with this notion, we provide extensive evidence that Thorase regulates mTORC1 in fibroblasts as well as neurons, suggesting our findings on Thorase inhibition of mTORC1 may be relevant to other tissues. In summary, our discovery that Thorase is a novel modulator of mTOR activity that acts through the disassembly of the mTORC1 sheds light onto how this pathway is terminated and provides a potential new therapeutic avenue to treat the myriad of human diseases related to dysregulated mTOR signaling.

## Methods

### Key resources

A list of resources used/generated in this study is provided as See Supplementary Table 2.

### Animals

All experimental procedures were according to the guidelines of Laboratory Animal Manual of the National Institute of Health Guide to the Care and Use of Animals and were approved by the Johns Hopkins Medical Institute Animal Care and Use Committee. Thorase-heterozygous C57BL/6 J mice (*ATAD1*^+/−^) generated in our previous studies^14,16^ were mated to obtain Thorase-knockout (KO) C57BL/6 J mice (*ATAD1*^−/−^) and littermates Thorase-wild type C57BL/6 J mice (*ATAD1*^+/+^). Genotypes were confirmed by PCR analysis on digested mouse tail samples. For all studies, we used female and male Thorase-knockout (KO) C57BL/6 J mice (*ATAD1*^−/−^) and littermates Thorase-wild type C57BL/6 J mice (*ATAD1*^+/+^). Because of the short lifespan of *ATAD1*^−/−^ mice (in this study, median survival of 24 days, please see Fig. 7i), we used animals between the ages p14-21 days for most biochemical studies. For the experiments where we injected animals with rapamycin or vehicle, we initiated triweekly injections at p10 onwards.

### Cell Culture

RagA^GTP/GTP^ mouse embryonic fibroblasts were a kind gift from Dr. Alejo Efeyan (CNIO, Spain); TSC2 KO/KO cells were a kind gift from Dr. David A. Kass (Johns Hopkins University). All mouse embryonic fibroblasts were maintained in Dulbecco’s Modified Eagle Medium (DMEM) plus 10% (v/v) bovine serum (FBS) and 1% Penicillin-Streptomycin, at 37 °C with a 5% CO_2_ atmosphere in a humidified incubator. Primary cortical neuron cultures (wild type, Thorase KO) were prepared from embryonic day 15 (E15) and 17 (E17) mouse pups, respectively in neurobasal media with B27 supplement (Gibco) and 1% Penicillin-Streptomycin in a humidified incubator^16^. Transfections were performed with Lipofectamine 2000 Reagent (Invitrogen) according to the manufacturer’s instruction.

### Recombinant proteins

All recombinant proteins were expressed in *E. coli* BL21(DE3) codon plus and induced by 1.0 mM isopropyl thiogalactoside (IPTG) at 16 °C overnight. The bacterial pellets were lysed using a microfluidizer (Microfluidics) in binding/ATPase buffer (100 mM Tris-HCl pH 7.5, 150 mM NaCl, 5 mM MgCl2, and 5% Glycerol) with protease inhibitors (Sigma) and centrifuged at 15,000 × *g* for 30 min. GST-tagged proteins were purified by using glutathione beads, respectively following manufacturer’s instructions. Protein samples eluted from the beads were further purified by size exclusive chromatography (GE Healthcare) using the ATPase buffer. The purity of the recombinant proteins was checked by Coomassie staining and western immunoblotting.

### Lentivirus Generation

Lentiviruses expressing Thorase were generated by the transient transfection of HEK293T cells with lentiviral plasmids described above using FuGENE HD Transfection Reagent. The cells were co-transfected with the Thorase lentiviral plasmids, the trans-complementation plasmids (pLP1 and pLP2), and the plasmid encoding the vesicular stomatitis virus envelope glycoprotein (VSVG) followed by sodium butyrate treatment 6 h after transfection^9,11^. The medium was replaced 24 h after transfection. Viral particles were harvested by collecting medium 48 h and 72 h after transfection and centrifuged at 3000 g for 10 min, then filtered through a 0.45 µm membrane. Viral particles were then concentrated by ultracentrifugation (2 h, 25,000 rpm, rotor SW28). The viruses were stored at −80 degrees until needed. The expression of Thorase was verified by infecting HEK293 cells with viruses and cells examined under fluorescence microscope 24–48 h after infection. Cells were harvested and lysates were resolved on SDS-PAGE and immunoblotted by probing with anti-Thorase.

### Recombinant protein purification

GST- tagged fusion proteins were expressed in *Escherichia coli* strain BL21-CodonPlus (DE3)-RIPL bacteria. Small cultures (100 ml) were grown overnight at 37 ^o^C and then transferred to 2-liter cultures for another 8 h. Cells were cold shocked at 4 ^o^C for 1 hour before adding 1 mM IPTG for overnight induction at 16 ^o^C^11^. Cells were lysed using micro-Fluidizer in binding buffer (1x phosphate buffer, pH 7.5, 150 mM NaCl, 2.5 mM MgCl2, 1 mM DTT, 5% glycerol) containing protease inhibitors and purified by using GST beads following the manufacturer’s instructions. To obtain untagged purified proteins, GST-tagged proteins bound to the GST beads were treated with Prescission protease to remove the GST tags. Samples were further purified using ion exchange chromatography and/or size-exclusion chromatography. Purity of the recombinant proteins was assessed by SDS-PAGE followed by Coomassie blue staining and immunoblotting.

### Pull down assays

For the identification of Thorase binding partners, recombinant GST-Thorase purified on beads was mixed with mouse whole brain cytosolic extract in the presence of non-hydrolysable ATP (ATPγS) in buffer A (50 mM HEPES, pH 7.5, 150 mM NaCl, 2.5 mM MgCl2, 1 mM DTT, 5% glycerol) and incubated with end-over-end mixing for 2 h. The beads were then extensively washed with buffer A containing ATPγS, and Thorase was cleaved from the GST beads with PreScission protease. The supernatant containing different protein complexes bound to Thorase was loaded into size-exclusion chromatography to separate the complexes. The different fractions eluted from the column were resolved by SDS-PAGE and individual bands (proteins) were excised for mass spectrometry analyses. Immunoblotting was used to confirm the presence of proteins identified by mass spectrometry.

Co-immunoprecipitation of endogenous Thorase and mTOR was carried out using lysates of whole mouse brains. Freshly isolated whole brains from wild type or Thorase KO mice were homogenized in buffer A containing protease inhibitors with or without 2 mM ADP, ATP or ATPγS^16^. Triton X-100 was added to a final concentration of 1% followed by rotation for 2 h at 4 ^o^C. Extracts were centrifuged at 15,000 g for 30 min and supernatant was incubated for 3 h at 4 °C with Protein G beads pre-bound with anti-Thorase or anti-mTOR antibodies. The beads were washed three times with buffer A plus 1 mM ADP, ATP or ATPγS and bound proteins were eluted from beads using 1x SDS-PAGE Laemmli buffer with DTT. The eluted proteins were resolved by SDS-PAGE. Immunoblotting analyzes were carried out with antibodies to Thorase, mTOR, Raptor, S6K and 4E-BP1.

For in vivo pull down of Thorase and mTOR, live cells were treated with or without 2.5 mg/ml DSP in the culture media and incubated for 30 min at 37 ^o^C^12,39^. The cells were washed three times with HBSS prior to harvesting. Cell lysates were prepared in the presence of 2 mM ADP, ATP, or ATPγS in buffer A and incubated with mixing for 2 h. The beads were then extensively washed with buffer A containing 1 mM ADP, ATP, or ATPγS. The eluted protein complexes were further separated on size exclusive chromatography (SEC). Fractions from SEC were resuspended in SDS-PAGE sample buffer and resolved by SDS-PAGE. Immunoblotting was used to confirm the presence protein complexes.

For in vitro pull down of Thorase and mTOR, recombinant GST-Thorase (wild type or variants) or GST-mTOR purified on beads were mixed with mouse whole brain lysates in the presence of 2 mM ADP, ATP, or ATPγS in buffer A and incubated with mixing for 2 h^11^. The beads were then extensively washed with buffer A containing 1 mM ADP, ATP, or ATPγS. The beads were resuspended in 1x SDS-PAGE sample buffer and eluted samples resolved by SDS-PAGE. Immunoblotting was used to confirm the presence of proteins bound to GST-tagged proteins. For assessing direct interaction between Thorase and mTOR, recombinant GST-Thorase purified on beads was mixed with purified non-tagged mTOR recombinant protein in the presence of 2 mM ATPγS in buffer A and incubated with mixing for 2 h. The beads were then extensively washed with buffer A containing 1 mM ATPγS. The sample eluted from the beads was loaded into size-exclusion chromatography to separate the complexes. The different fractions eluted from the column were resolved by SDS-PAGE to confirm the presence of GST-Thorase and mTOR. Mass spectrometry was conducted at Harvard Medical School Taplin Spectrometry Facility.

### Starvation and mTORC1 activation experiments

Starvation and stimulation in HEK-293T cells and MEFs was performed as previously described^7,39,56^, with some modifications as follows. Cells cultured in complete DMEM (supplemented with glutamine, FBS and PS) were rinsed with and incubated in amino acid-free HBSS supplemented with dialyzed FBS (amino acid free), glucose, glutamine, and PS (CCM media) for 50 min and stimulated with a 10X mixture of amino acids in CCM for 15 min. Cells were fixed with 4% PFA or harvested at 5, 10. 20, 30 or 50 min after amino acid stimulation for 10 min.

For serum starvation, subconfluent cells were starved in DMEM for 16 h and stimulated with insulin for 15 min, similar to described by Menon and colleagues^41^, and Hoxhaj and colleagues^40^.

For live imaging experiments, cells co-expressing a combination of Thorase-GFP, Thorase-RFP, mTOR-YFP, Lamp1-GFP, Lamp1-RFP and/or Mito-RFP cultured in CCM media were rinsed with and incubated in amino acid-free HBSS supplemented with glucose, glutamine, and PS for 50 min (HEK293) or 2 h (MEFs). Media was then replaced with a 10X mixture of amino acids in CCM and live imaged immediately using a Zeiss LSM 5 Duo confocal microscope with continuously perfused at a rate of 1 ml/min with CCM media with 10X mixture of amino acids to monitor co-localization of Lamp1-RFP and Thorase-GFP or mTOR-YFP. Cells were imaged for 3 min (1 image/15 sec) or 30 min (1 image/min). All images were analyzed using NIH ImageJ software (Rasband, W.S., NIH, http://rsb.info.nih.gov/ ij/, 1997–2007).

### Immunostaining

Wild type and Thorase KO mice were anesthetized with sodium pentobarbital, perfused with 4% PFA in PBS (phosphate buffer, pH 7.4), and immediately decapitated and the brains removed^14,16^. Post fixation was performed in 4% PFA in PBS overnight and fixed brains were then dehydrated in 30% glucose for another 48 h. Brains were sectioned on a microtome at 30 μm, washed in PBS and then permeabilized with 0.3% Triton X-100 in PBS containing 5% goat serum for 1 h at room temperature. After three 5-minute washes with PBS, sections were incubated with primary antibodies in PBS containing 0.1% Triton X-100, 0.01% sodium azide and 2.5% goat serum overnight at 4 °C. Following washout with PBS, sections were incubated with secondary antibodies 3 h at room temperature. Sections were washed three times with PBS and cell nuclei counterstained with DAPI (Invitrogen, Molecular Probes). Sections were mounted on slides and a Zeiss LSM 880 laser-scanning confocal microscope was used to acquire images under identical acquisition parameters for side-by-side comparison. For co-localization experiments, we defined mTOR puncta as any mTOR accumulation at the lysosome that was at least 1 µm in size. 3D images were generated and analyzed by Imaris 9.2.0 (Bitplane, AG www.imaris.com). For in vitro cultures grown on coverslips and fixed in 4% PFA, immunostaining followed the same protocol as described above.

### De novo protein synthesis assessment

Fibroblasts derived from wild type or Thorase KO mouse embryos were grown in 12 well plates (Falcon) to around 80% confluency and pre-treated with vehicle DMSO or 500 nM rapamycin if needed^39^. For ^35^S-labeling assays, 25 μCi of EasyTag™ EXPRESS^35^S Protein Labeling Mix (Perkin Elmer) per well were added to the media, and cells were incubated at 37 ^o^C and 5% CO_2_ for 1 hour. Cells were then washed twice with cold PBS and lysed in extraction buffer (2% NP-40, 50 mM Tris-HCl, 150 mM NaCl, 5 mM EGTA, protease inhibitor cocktail), spun down and supernatants collected. Protein was concentrated with methanol/heparin, and protein pellets then resuspended in 8 M Urea/150 mM Tris (pH 8.5). Samples were then analyzed in a LS 6500 Multi-purpose Scintillation Counter (Beckman Coulter), and radioactive counts normalized to sample protein concentration as measured by Pierce BCA protein assay (Thermo Scientific). A fraction of the precipitated protein was run in 4–20% SDS-PAGE gels and the emitted radioactivity was images using Typhoon Imager (Amersham).

For SUnSET assays, per every milliliter of media 10 µg of Puromycin (Gibco), and in control conditions 100 ug of cycloheximide (Sigma) additionally, was added and cells were incubated at 37 ^o^C and 5% CO_2_ for 20 min. The collected protein lysates were run in 4–20% SDS-PAGE gels and the extent of newly synthetized proteins were detected using an anti-puromycin antibody.

### SiMPull experiments

HEK293 cells were transiently co-transfected with YFP-mTOR, and mCherry-Raptor followed by cell lysis in lysis buffer (40 mM Hepes, pH 7.5, 120 mM NaCl, 10 mM sodium pyrophosphate, 10 mM β-glycerophosphate, 1X protease inhibitor mixture and 0.3% CHAPS). YFP-mTOR- and mCherry-Raptor-only lysates were obtained by transiently transfecting cells with YFP-mTOR and mCherry-Raptor, respectively. Lysates were centrifuged at 12,000 × g for 12 min at 4 °C and diluted 150-fold to obtain a surface density optimal for single-molecule analysis (~800 molecules in 5000 mm^2^ imaging area)^28^. For control experiments, HEK293 cells were transiently transfected with PKA isoforms PKA-RIIβ-Flag-mCherry and PKA-Cα-HA-YFP. After 24 h of expression, cells were lysed using a buffer containing 10 mM Tris pH 7.5, 1% NP-40, 150 mM NaCl, 1 mM EDTA, 1 mM benzamidine, 10 μg/ml leupeptin, 1 mM NaF, 1 mM Na_3_VO_4_. The lysate thus obtained was centrifuged at 14,000 × g for 20 min and subsequently used for SiMPull^28,57^. Recombinant Thorase was incubated with 20-fold molar excess of Cy5-maleimide dye at room temperature for an hour and at 4 ^o^C overnight. Unreacted dyes were removed by gel filtration using Zeba spin desalting columns.

Single-molecule experiments were performed on a prism-type TIRF microscope equipped with an electron-multiplying CCD camera (EM-CCD)^28^. For single-molecule pull-down experiments, quartz slides and glass cover slips were passivated with 5000 MW methoxy poly-(ethylene glycol) (mPEG, Laysan Bio) doped with 2-5% 5000 MW biotinylated PEG (Laysan Bio). Cell lysates were pulled down with biotinylated antibodies against YFP or RFP, already immobilized on the surface via neutravidin-biotin linkage. Thorase interactions with mTORC1 and its individual components were studied by incubation of pulled-down mTORC1 or its individual components with a pre-determined concentration of Cy5-Thorase in T200-BSA buffer supplemented with 5 mM AMP-PNP (or ATP), 20 mM MgCl_2_ and 10% Glycerol. 15 frames were recorded from each of 20 different imaging areas (5,000 μm^2^) and isolated single-molecule peaks were identified by fitting a Gaussian profile to the average intensity from the first ten frames. Mean spot-count per image for YFP and mCherry was obtained by averaging 20 imaging areas using MATLAB scripts.

Co-localization data were acquired from two or three separate movies from the same region of a slide using YFP, m-Cherry and Cy5 excitation. The co-localization criterion was set to a diffraction limited region of ~300 nm, which corresponds to 2 pixels for this TIRF setup. % co-localization was calculated as the % molecules co-aligned with the pulled down component, unless otherwise stated. YFP, mCherry and Cy5 images taken from different areas were also overlapped to determine the probability of false co-localization arising from random spatial overlap of single molecules. Average % co-localization was calculated based on a minimum of 30 individual slide areas. For photobleaching analysis, single-molecule fluorescent time traces from individual YFP or mCherry spots were manually scored for the number of photobleaching steps and the stoichiometry of the molecules was assessed^28,29,57^. Briefly, the fluorescence trace of each molecule was classified as having one to four bleaching steps or was discarded if no clean bleaching steps could be identified. The intensity of molecules scored as bleaching in one and two steps was plotted to verify scoring: on average we expect the fraction of molecules bleaching in two steps to be twice as bright as the molecules bleaching in one step. The intensity of discarded molecules was also plotted to ensure unbiased scoring as observed via lack of enrichment of any specific intensities. To convert the photobleaching step distribution to monomer/dimer fraction, the percentage of molecules bleaching in two steps was compared with a calibration experiment. This conversion was performed only when >90% of the molecules bleached in one or two photobleaching steps.

### Cell migration (scratch) assay

Wild type or Thorase KO fibroblasts were grown in tissue culture-treated glass bottom 12 well plates (Corning™ Costar™) to around 70% monolayer confluency. A sterile p200 pipet tip was used to scrape the cell monolayer in a straight line to create a “scratch”. The media with the cell debris were replaced with fresh growth medium containing vehicle DMSO or 500 nM rapamycin^39^. Live imaging of the scratched regions was performed using EVOS®-FL-Auto phase-contrast microscope in a tissue culture incubator at 37 °C with 5% CO_2_. Cells were imaged for 8 h (1 image/15 min). All images were analyzed using NIH ImageJ software (Rasband, W.S., NIH, http://rsb.info.nih.gov/ ij/, 1997–2007). For each image, distances between one side of scratch and the other were measured (nm) and rate of cell migration were determined by dividing the initial scratch width by the time spent in migration (time point to obtain scratch closure)^58,59^.

### Rapamycin treatment in mice

Thorase homozygous KO mice and wild type littermates were obtained by crossing heterozygous Thorase mice^14,16^. Animal experiments were performed in compliance with the regulations of the Animal Ethical Committee of the Johns Hopkins University Animal Care and Use Committee. Wild type or Thorase KO mice were injected intraperitoneally with 6mk/kg Rapamycin (#R-5000, LC laboratories) or vehicle (10% PEG400, 10% Tween 80 in water), three times per week starting at 10–14 days postnatally.

### Study design and reproducibility

All biochemical and imaging experiments were repeated independently at least three times. For biochemical experiments involving brain tissue or mouse embryonic fibroblasts, three biological replicates per group were used per experiment. For mouse survival experiments and immunohistochemical analysis, enough sample size (as determined via post hoc power analysis) was used, and mice pertaining to different litters were used. We found our results reproducible when experiments were performed by two independent researchers. For sample randomization, for biochemical experiments where two treatments were applied, two technical replicates per biological sample (three biological replicates total per group) were used. For mouse experiments where either vehicle or rapamycin was injected, an independent researcher randomly allocated mice to either treatment group. Regarding blinding during data collection, for immunofluorescence, immunohistochemistry and electron microscopy imaging, researchers were blinded to genotypes. For mouse survival analysis, researchers were blinded to both genotype and treatment.

### Data analysis and statistics

All experiments were repeated at least three times and quantitative data are presented as the mean ± standard error of the mean (SEM) by GraphPad prism6 software (Instat, GraphPad Software). Statistical significance was assessed by ANOVA. The significant differences were identified by post-hoc analysis using the Tukey–Kramer post-hoc method for multiple comparisons. Assessments were considered significant with a *p* < 0.05 and non-significant with a *p* > 0.05 (Table S2). To assess the sufficiency of sample size, post hoc power analysis was performed using GraphPad Prism, considering a power greater than 0.9 sufficient.

### Reporting summary

Further information on research design is available in the Nature Research Reporting Summary linked to this article.

## Supplementary information

Supplementary Information

Reporting Summary

## Data Availability

Source data are provided with this paper. There are no restrictions on data availability in this manuscript. The data in main and supplementary figures generated in this study are provided in the Supplementary Information as a Source Data file. All raw data and source files are available via DRYAD 10.5061/dryad.6wwpzgn25. Mass spectrometry data generated for this study is available via the ProteomeXchange repository with the following accession number, MSV000089776. Source data are provided with this paper.

## Source data

Source Data
