# Supplementary material for: AAA + ATPase Thorase inhibits mTOR signaling through the disassembly of the mTOR complex 1
Source: Nat Commun. 2022 Aug 17;13:4836. doi: 10.1038/s41467-022-32365-2 (PMC9385847; doi:10.1038/s41467-022-32365-2)
Supplement: Supplementary file 1 — Supplementary Information [file 41467_2022_32365_MOESM1_ESM.pdf]

AAA+ ATPase Thorase Inhibits mTOR Signaling Through the Disassembly of the mTOR Complex 1

George K.E. Umanah, Leire Abalde-Atristain, Mohammed Repon Khan, Jaba Mitra, Mohamad Aasif Dar, Melissa Chang, Kavya Tangella, Amy McNamara, Samuel Bennett, Rong Chen, Vasudha Aggarwal, Marisol Cortes, Paul F. Worley, Taekjip Ha, Ted M. Dawson* and Valina L. Dawson*

Supplementary Figures 1 -7

Supplementary Tables 1-2

Source Data

-Supplementary Data file (raw data) in separate Excel file

Supplementary Figure 1

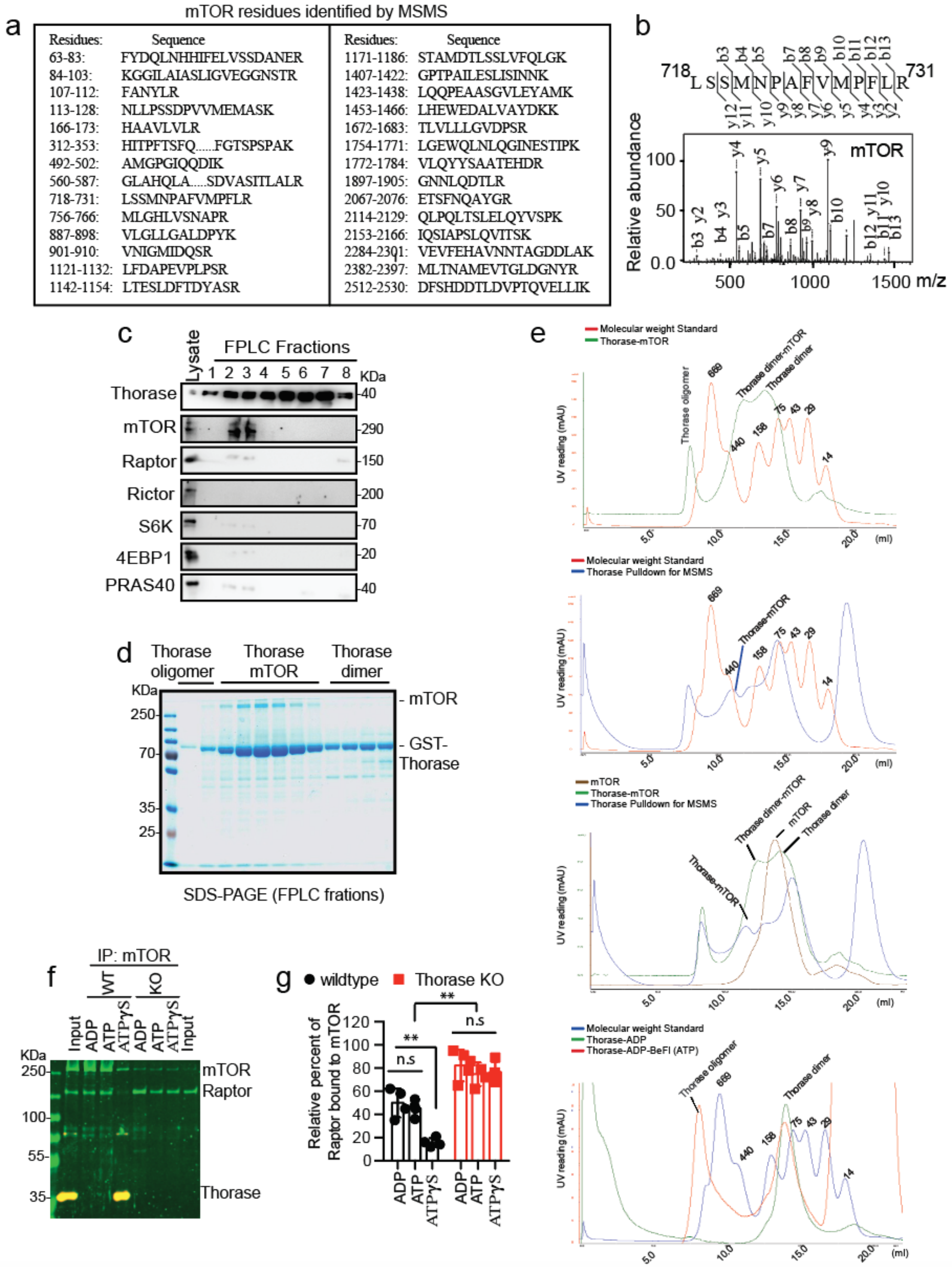

Supplementary Figure 1. Thorase interaction with mTOR is concomitant to a decrease in binding of mTOR to other mTORC1-related proteins

a, A list of mTOR peptides identified by mass spectrometry in Thorase pulldown. **b**, Representative mTOR peptide fragmentation detected by mass spectrometry after Thorase pulldowns. **c**, Immunoblots of FPLC fractions Thorase pulldown of mTOR. **c**, FPLC profile confirming direct interaction of purified recombinant mTOR and GST-Thorase complex on FPLC size-exclusive column. **d**, An image of Coomassie stained SDS-PAGE of fractions from FPLC shown in C. **e,** FPLC profiles of Thorase-mTOR, mTOR or Thorase alone complexes. *Second panel*, Overlap of protein molecular weight standards (red) and purified recombinant GST-Thorase/mTOR proteins (green) FPLC profiles. Peaks corresponding to GST-Thorase/mTOR complexes are indicated and as shown in D. *Top panel*, Overlap of protein molecular weight standards (red) and purified recombinant Thorase pulldown of protein complexes from Thorase KO lysates (blue) FPLC profiles. Peak corresponding to Thorase-mTOR complexes is indicated and as shown in C (FPLC fractions 2 and 3). *Third panel*, Overlap of purified recombinant mTOR alone (brown), Thorase pulldown of protein complexes (blue) and purified Thorase-mTOR (red) FPLC profiles. *Fourth panel*, Alignment of protein molecular weight standards (blue), purified recombinant Thorase with ADP (green) and purified recombinant Thorase with ATP analog, ADP-BeFl (red) FPLC profiles. Peaks corresponding to Thorase dimers and oligomers are labeled. **f**, Immunoblot images of mTOR pulldown from wildtype (WT) or Thorase knockout (KO) MEFs in the presence of different nucleotides. **g,** quantification of blots in F (n=4 independent pulldowns). Data is mean ± standard error of the mean [SEM] of experiments performed, *p < 0.05, **p < 0.01, n.s p > 0.05, two-way ANOVA with Tukey's post-hoc test (exact p-values indicated in Data Source File).

Supplementary Figure 2

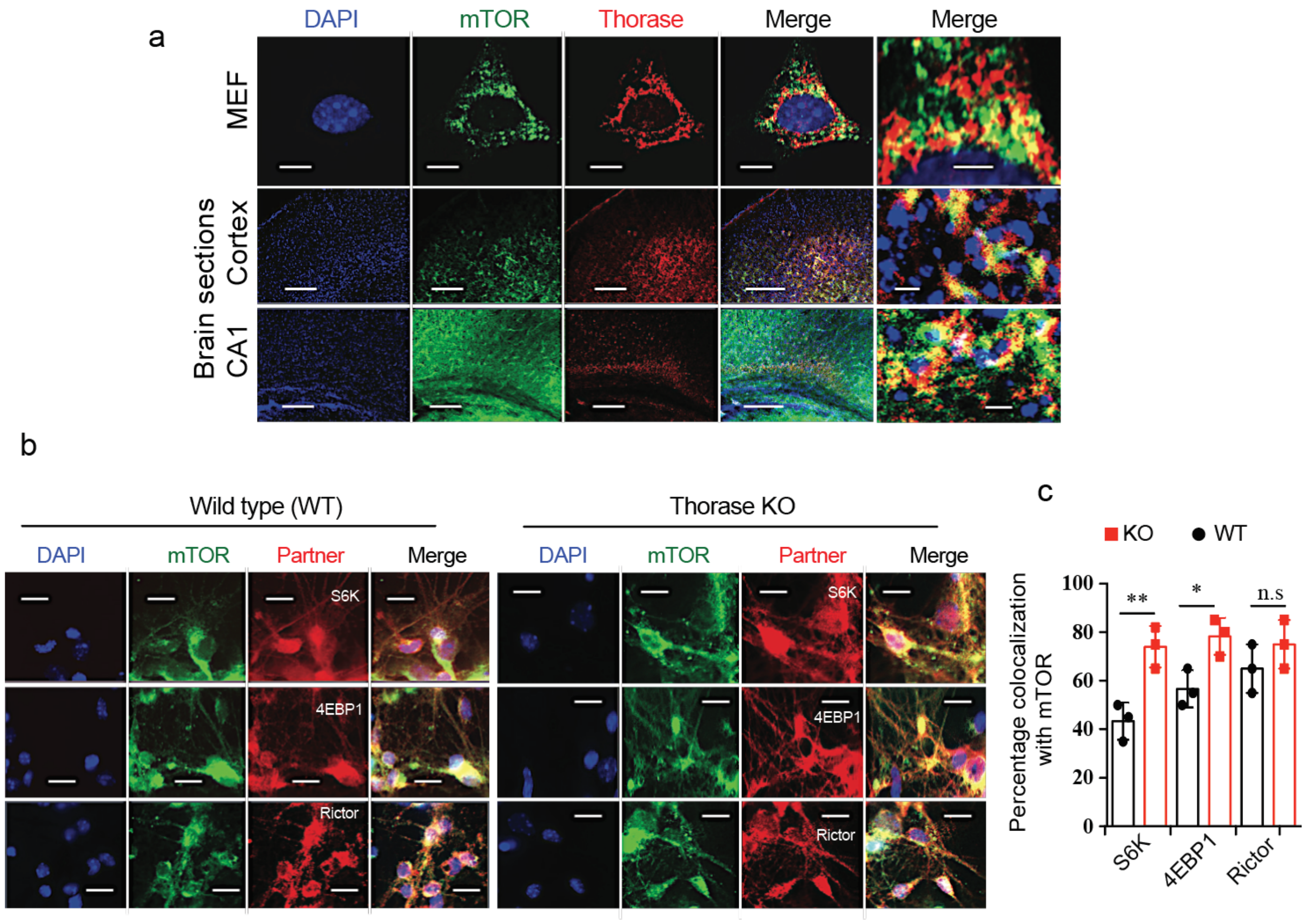

Supplementary Figure 2. Increased mTOR colocalization with mTORC1 proteins in Thorase knockout mice and fibroblasts

a, Representative images of mouse embryonic fibroblasts (MEF), primary cortical neurons, mouse brain cortex and hippocampus CA1 showing colocalization of Thorase and mTOR. Scale bars are (MEF: 20µm and 2µm, Cortex/CA1: 200µm and 20µm). **b**, Representative images of colocalization of mTORC1 proteins with mTOR in WT and KO primary cortical neurons. Scale bar is 20µm. **c**, Quantification of colocalization of images in B (n=3 independent experiments). Data is mean ± standard error of the mean [SEM] of experiments performed, *$p < 0.05$, n.s $p > 0.05$, analyzed with two-tailed unpaired t-tests (exact p-values indicated in Data Source File).

Supplementary Figure 3

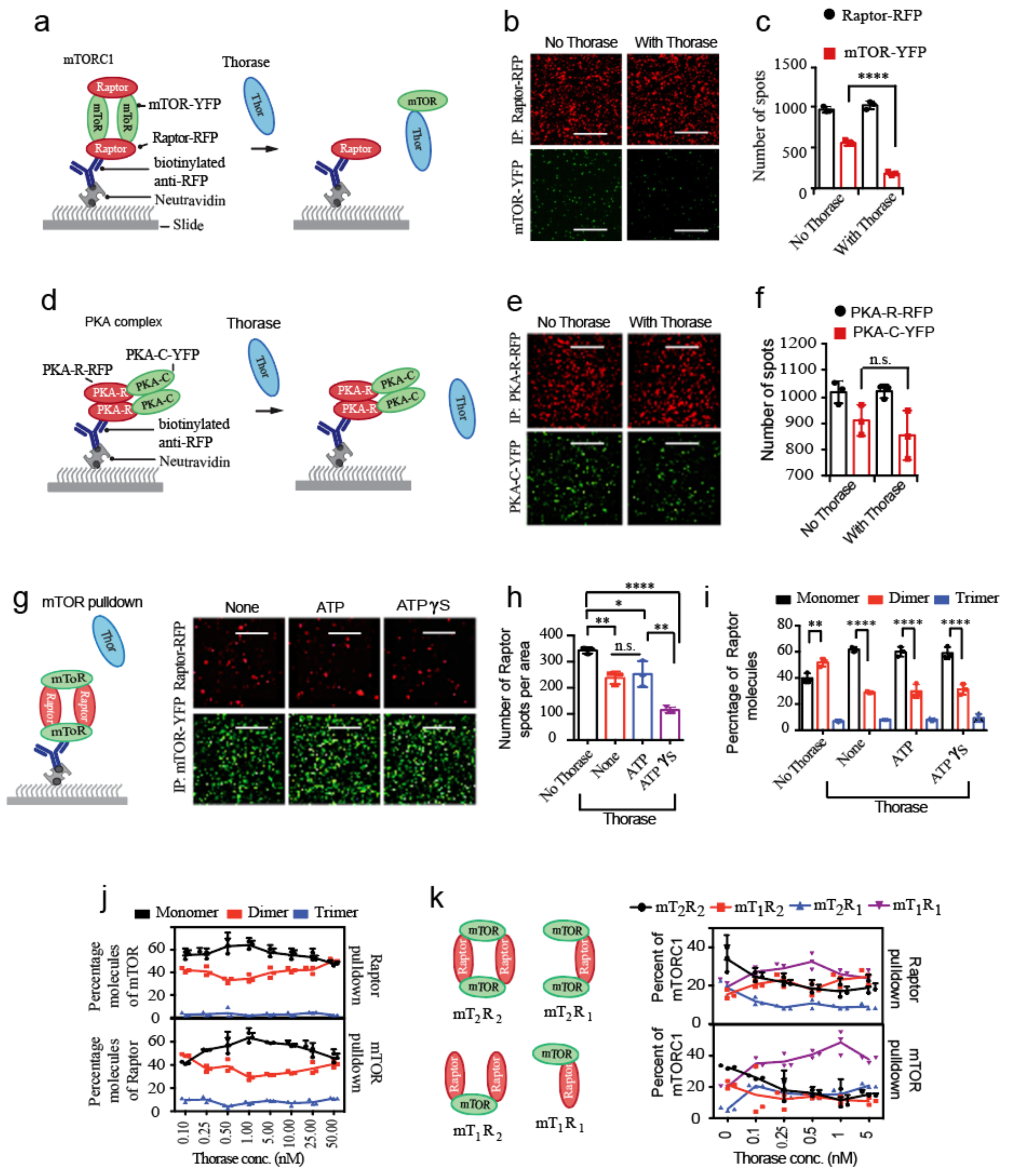

Supplementary Figure 3. ATP binding is necessary for Thorase disassembling of the mTOR complex

a, Schematic diagram of SiMPull analyses of mTOR-Raptor complex in the presence of Thorase. Adapted from Jain et al., [28]. **b**, Representative images from mTORC1 SiMPull confirming Thorase disassembles the mTOR-Raptor complex. Scale bars are 10 µm. **c**, Quantification of images in C (n = 3 independent SiMPull assays). **d**, Schematic diagram of SiMPull analyses of PKA complex (control) in the presence of Thorase. Adapted from Jain et al., [28]. **e**, Representative images from PKA SiMPull showing no significant disassembly of the control PKA complex in the presence of Thorase. Scale bars are 10 µm. **f**, Quantification of images in E (n = 3 independent SiMPull assays). Data in D, F, H, I, J and K are mean ± standard error of the mean [SEM] of experiments performed, ****$p < 0.0001$, ***$p < 0.001$, *$p < 0.05$, n.s $p > 0.05$, ANOVA with Tukey-Kramer post-hoc test compared with control (no Thorase). **g**, Schematic diagram, and representative images of Raptor-pulldown SiMPull analyses of mTOR-Raptor complex in the presence of Thorase and different nucleotides. Adapted from Jain et al., [28]. Scale bars are 10 µm. **h**, Quantification of number of mTOR spots per imaging area in G (n = 3 independent SiMPull assays). **i**, Quantification of the amount of different species of mTOR in the presence of Thorase and different nucleotides (n = 3 independent SiMPull assays). **j**, Graphical representation of the disassembly of different species of mTOR and Raptor in the presence of different concentrations of Thorase (n = 3 independent SiMPull assays). **k**, Schematic diagram, and graphical representation of different complexes of mTORC1 in the presence of different concentrations of Thorase (n = 3 independent SiMPull assays). Data in B and C are mean ± standard error of the mean [SEM] of experiments performed, ****$p < 0.0001$, ***$p < 0.001$, *$p < 0.05$, n.s $p > 0.05$. c,f: two-tailed unpaired t-tests. h, i: one-way ANOVA with Tukey's post-hoc test (exact p-values indicated in Data Source File).

Supplementary Figure 4

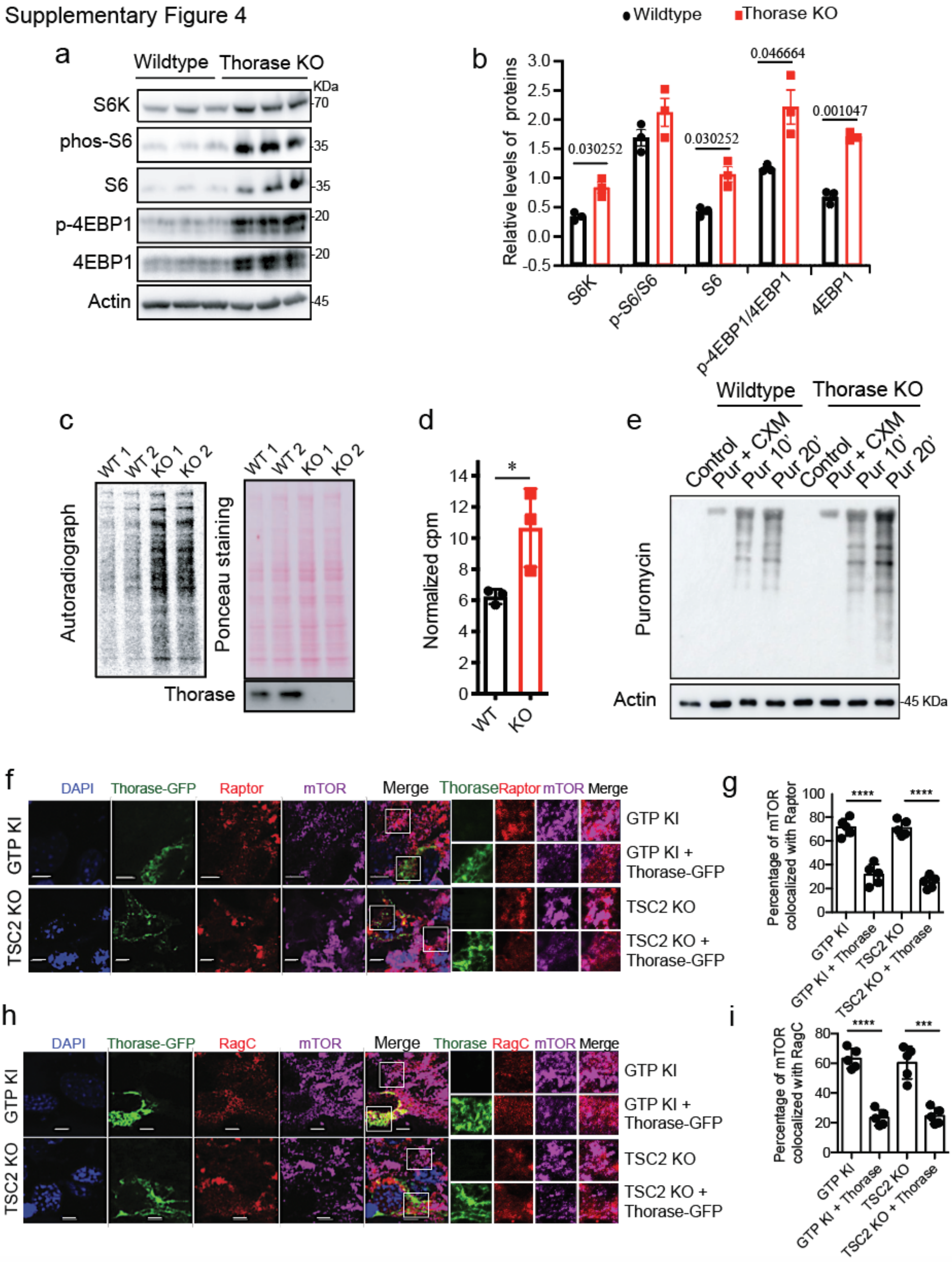

Supplementary Figure 4. mTORC1- related protein levels are elevated in Thorase knockout mice and fibroblasts

a, Immunoblot images of mTORC1 protein expression in wildtype (WT) and Thorase knockout (KO) MEFs. **b**, Graphical representation of quantified blots in a (n = 3 independent experiments). **c**, Representative autoradiograph and ponceau staining showing increased protein synthesis (MetS^{35} incorporation) in KO MEFs compared to WT MEFs. **d**, Graphical representation of blots in C (n = 3 independent experiments). **e**, Representative images showing increased protein synthesis in (puromycin incorporation) in KO MEFs compared to WT MEFs. Data in B and D are mean ± standard error of the mean [SEM] of experiments performed, ***p < 0.001, *p < 0.05, n.s p > 0.05, B: Data from unpaired t test corrected for multiple comparisons with Holm-Šídák method. D: data from unpaired t test. G-I: ANOVA with Tukey-Kramer post-hoc test compared with WT. **f-i,** Representative images of GTP KI or TSC2 KO mouse embryonic fibroblasts expressing Thorase-GFP (green). **f,** Cells were immunostained to evaluate mTOR(purple)-Raptor (red) co-localization. Scale bars are 5 µm. **g,** Quantification of mTOR-Raptor co-localization of images in A (n=5 independent experiments). **h,** Cells were immunostained to evaluate mTOR(purple)-RagC (red) co-localization. Scale bars are 5 µm. **i,** Quantification of mTOR-RagC colocalization of images in C (n=5 independent experiments). Data are mean ± standard error of the mean [SEM] of experiments performed, **p < 0.01, n.s p > 0.05. b: multiple unpaired t tests with Holm-Šídák test for multiple comparisons. d: two-tailed unpaired t-test. d, g, i: two-tailed unpaired t-tests (exact p-values indicated in Data Source File).

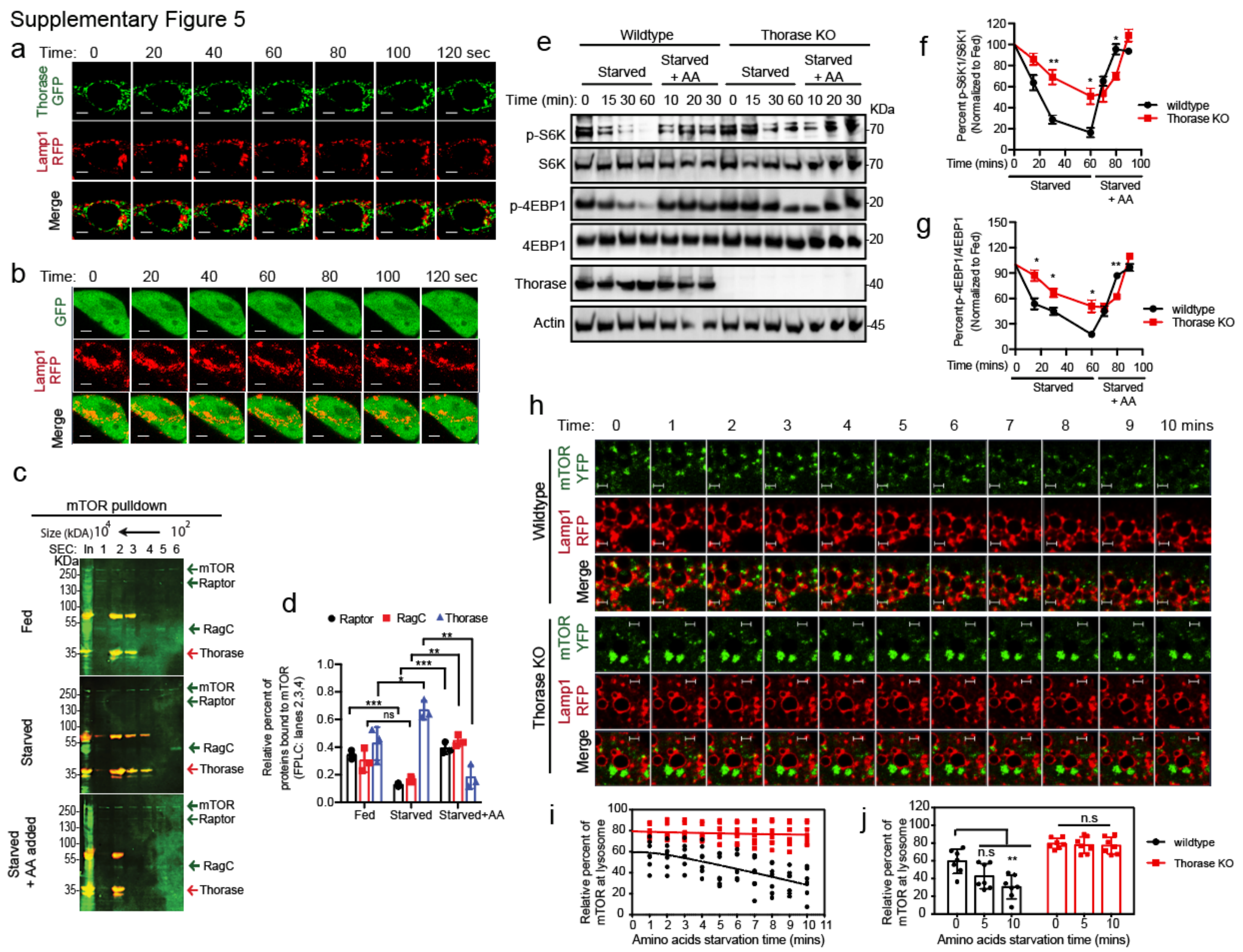

Supplementary Figure 5. Thorase colocalizes with lysosome, and modulates mTORC1 activity

a-b, Representative images from live imaging of cells co-expressing Thorase-GFP (**a**) or control GFP alone (**b**) with Lamp1-RFP. Scale bars are 5 µm. **c,** Representative immunoblot images of mTOR IP (pulldown) protein complexes from WT MEFs under different nutrient statuses and protein complexes separated by size exclusive chromatography (SEC). Lanes: In (total lysate input), 1-6 are SEC fractions. **d,** Quantification of blots in **a** showing the relative amount of Thorase, Raptor and RagC bound to mTOR (n=3 independent pulldowns). **e-g**, Representative immunoblots of WT and KO MEFs showing mTORC1 activation (phosphorylation) status during amino acids (AA) starvation and/or supplementation at different times (n=3 independent experiments). **h**, Representative images from live imaging of wildtype or Thorase knockout (KO) MEFs co-expressing

mTOR-GFP with Lamp1-RFP during amino acids starvation. Scale bars are 2 µm. **i**, Graphical representation of the percent of mTOR at the lysosome during amino acids starvation from live imaging in **c** (n = 7 independent experiments). **j**, Graphical representation of selected time points from live imaging in c (n = 7). Data in D and E are mean ± standard error of the mean [SEM] of experiments performed, $^{**}p < 0.01$, n.s $p > 0.05$. d, j: one-way ANOVA with Tukey's post-hoc test. f, g: two-tailed unpaired t-tests (exact p-values indicated in Data Source File).

Supplementary Figure 6

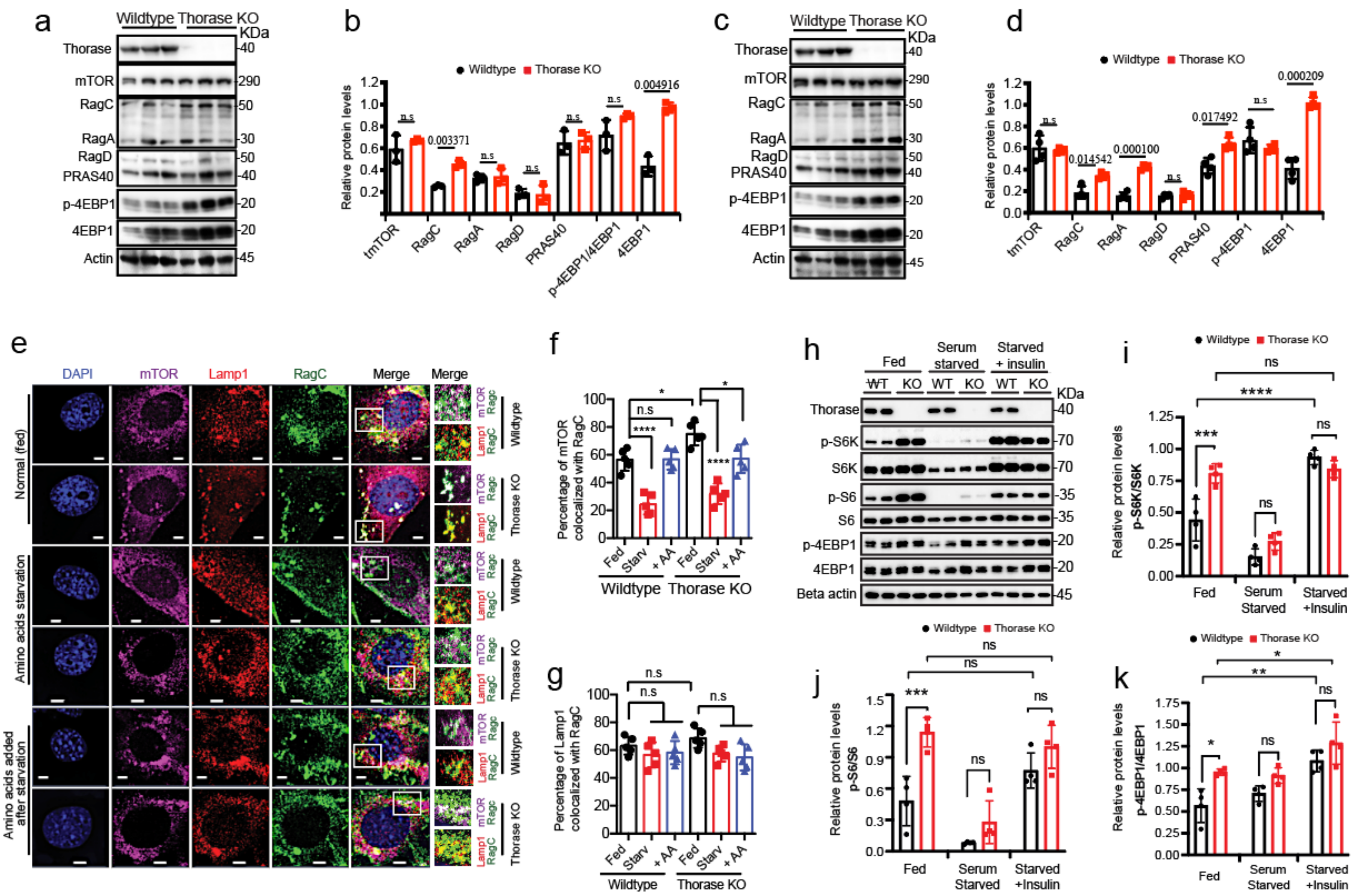

Supplementary Figure 6. Lack of Thorase results in increased RagC levels, without alterations in their localization or binding to mTOR, and Thorase knockout cells respond normally to serum starvation

a, Immunoblot images of mTORC1 pathway proteins in wild type (WT) and Thorase knockout (KO) mouse brain lysates. **b,** Quantification of samples in A (n = 3 biologically independent samples). **c**, Immunoblot images of mTORC1-associated proteins in wild type (WT) and Thorase knockout (KO) mouse embryonic fibroblast lysates. **d,** Quantification of samples in C (n = 3 independent experiments). **e,** Representative images of wild type and Thorase KO MEFs under different nutrient status showing mTOR(purple)-RagC (green) and Lamp1(red)-Ragc(green) co-localization. Scale bars are 5 µm. **f,** Quantification of mTOR-RagC colocalization of images in A (n=5 independent experiments). **g,** Quantification of Lamp1-RagC colocalization of images in A (n=5 independent experiments). **h,** Representative blots of mTORC1 effector levels upon 16 h serum starvation and 15-minute re-stimulation with insulin, and accompanying quantification of relative protein levels of **i,** p-

S6K; **j,** p-S6 and **k,** p-4EBP1. i-k data are mean ± standard deviation [SD] of n= 4 independent experiments, ***$p < 0.001$, n.s $p > 0.05$. b, d: multiple unpaired t tests with Holm-Šídák test for multiple comparisons. f, g, i, j, k: 2-way ANOVA with Tukey's post-hoc test (exact p-values indicated in Data Source File).

Supplementary Figure 7

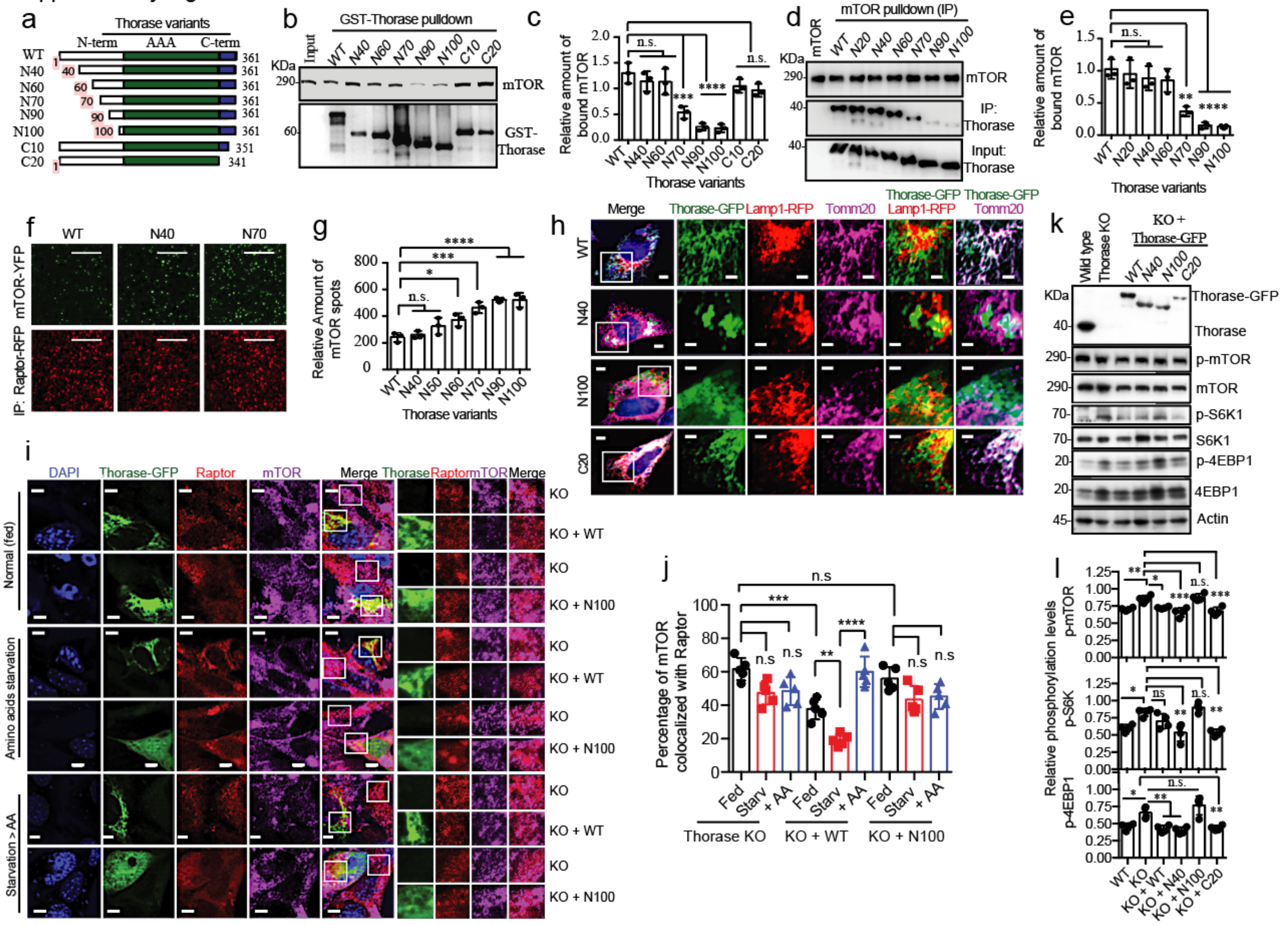

Supplementary Figure 7. Thorase N-terminus is essential for the interaction and regulation of mTOR activity

a, A schematic diagram of truncated variants of Thorase. **b**, Immunoblot images of different GST-Thorase truncated variants pulldown of mTOR. **c**, Quantification of blots in B (n = 3 independent pull downs). **d**, Immunoblot images of mTOR pulldown of different Thorase truncated variants. **e**, Quantification of blots in D (n = 3 independent pull downs). **f,** Representative images from Raptor-pulldown SiMPull in the presence of Thorase truncated variants. Scale bars are 10 µm. **g**, Quantification of signal intensities of images in f (n = 3 independent SiMPull experiments). **h**, Representative images of Thorase knockout (KO) mouse embryonic fibroblasts (MEFs) expressing Lamp1-RFP and Thorase-GFP truncated variants. Scale bars are 10 µm (low), 5µm (high) magnification. **i,** Representative images of Thorase KO mouse embryonic fibroblasts expressing

Thorase-GFP (green) wildtype (WT) or N-terminal truncated mutant (N100). Scale bars are 5 μm. Cells under different nutrient status were immunostained to evaluate mTOR(purple)-Raptor (red) co-localization. **j,** Quantification of mTOR-Raptor colocalization of images in a (n=5 independent experiments). Data are mean ± standard error of the mean [SEM] of experiments performed, $^{**}p < 0.01$, ANOVA with Tukey-Kramer post-hoc test. **k,** Immunoblot images of mTORC1 proteins in lysates from Thorase-KO MEFs expressing different Thorase-GFP truncated variants. **l,** Quantification of the relative amount of phosphorylated mTOR (pmTOR), S6K (pS6K) and 4EBP1 (p4EBP1) from blots in **a** (n = 4 independent experiments). Data are mean ± standard error of the mean [SEM] of experiments performed, $^{*}p < 0.05$, n.s $p > 0.05$, c, e, g, l: one-way ANOVA with Tukey's post-hoc test. j: two-way ANOVA with Tukey's post-hoc test (exact p-values indicated in Data Source File).

Supplementary Tables

Supplementary Table 1: List of proteins identified in Thorase pulldown of mouse brain cytosolic extract

	Number of samples	Unique peptide	Total peptide	Gene Symbol	Avg. Precursor Intensity
1	3	165	197	Dync1h1	1.35E+06
2	3	162	218	Sptan1	2.50E+06
3	3	127	150	Sptbn1	1.66E+06
4	3	80	97	Map2	1.25E+06
5	2	61	71	Spnb3	1.03E+06
6	3	53	65	Map1a	1.26E+06
7	3	50	57	Tln1	8.60E+05
8	3	48	52	Itpr1	1.00E+06
9	3	47	51	Dmxl2	1.07E+06
10	3	45	46	Plec	6.73E+05
11	3	41	45	Map1b	8.85E+05
12	3	41	43	Ank2	1.15E+06
13	3	38	40	Tln2	8.78E+05
14	3	37	41	Spta1	9.14E+05
15	3	29	31	Cltc	8.41E+05
16	3	29	29	Myo18a	7.89E+05
17	3	28	33	Tnr	1.27E+06
**18**	**3**	**28**	**30**	**Mtor**	**6.51E+05**
19	3	28	30	Fasn	6.57E+05
20	3	24	26	Cntn1	9.94E+05
21	3	22	25	Bsn	5.91E+05
22	3	22	25	Zzef1	6.06E+05
23	3	20	20	Vps13c	6.32E+05
24	3	19	25	Cntnap1	1.21E+06
25	3	19	19	Nbea	7.26E+05
26	3	17	19	Dnm1	7.04E+05
27	3	16	21	Atp1a1	7.10E+05
28	3	15	16	Vps13a	7.47E+05
29	3	15	16	Ank1	8.79E+05
30	3	15	15	Myh9	5.72E+05
31	3	15	15	Usp9x	9.65E+05
32	3	14	27	Atad1	2.51E+06
33	3	14	17	Nf1	4.76E+05
34	3	14	14	Htt	4.65E+05
35	3	13	20	Tuba1a	1.40E+06

Supplementary Table 2: List of key resources

KEY RESOURCES TABLE

REAGENT or RESOURCE	SOURCE	IDENTIFIER
Antibodies		
Mouse monoclonal anti-Thorase (1:50 dilution)	NeuroMab	Cat#75-157; RRID:AB_2290002
Rabbit monoclonal anti-mTOR (1:1000-1:2000 dilution)	Cell Signaling Technologies	Cat#2983
Rabbit monoclonal anti-phos-S2448 mTOR (1:1000-1:2000 dilution)	Cell Signaling Technologies	Cat#5536
Rabbit monoclonal anti-phos-S2481 mTOR (1:1000-1:2000 dilution)	Cell Signaling Technologies	Cat#2974
Rabbit monoclonal anti-Raptor (1:1000-1:2000 dilution)	Cell Signaling Technologies	Cat#2280
Rabbit monoclonal anti-phos-S792 Raptor (1:1000-1:2000 dilution)	Cell Signaling Technologies	Cat#2083
Rabbit monoclonal anti-Rictor (1:1000-1:2000 dilution)	Cell Signaling Technologies	Cat#2114
Rabbit monoclonal anti-p70 S6K (1:1000-1:2000 dilution)	Cell Signaling Technologies	Cat#2708
Rabbit monoclonal anti-phos-T389 p70 S6K (1:1000-1:2000 dilution)	Cell Signaling Technologies	Cat#9205
Rabbit monoclonal anti-anti-S6 (1:1000-1:2000 dilution)	Cell Signaling Technologies	Cat#2217
Rabbit monoclonal anti-phos-S240/244 S6 (1:1000-1:2000 dilution)	Cell Signaling Technologies	Cat#2215
Rabbit monoclonal anti-4EBP1 (1:1000-1:2000 dilution)	Cell Signaling Technologies	Cat#9644
Rabbit monoclonal anti-phos-T37/46 4EBP1 (1:1000-1:2000 dilution)	Cell Signaling Technologies	Cat#2855
Rabbit monoclonal anti-eIF4B (1:1000-1:2000 dilution)	Cell Signaling Technologies	Cat#3592
Rabbit monoclonal anti-ULK1 (1:1000-1:2000 dilution)	Cell Signaling Technologies	Cat#8054
Rabbit monoclonal anti- phos-S757 ULK1 (1:1000-1:2000 dilution)	Cell Signaling Technologies	Cat#6888
Rabbit monoclonal anti-Rheb1 (1:1000-1:2000 dilution)	Cell Signaling Technologies	Cat#13879
Anti-rabbit IgG-HRP conjugate secondary (1:2000 dilution)	Abcam	Cat# ab98467; RRID:AB_10674445
Anti-mouse IgG-HRP conjugate secondary (1:2000 dilution)	Abcam	Cat# ab6823 RRID:AB_955395
Mouse anti-β-actin-HRP (1:10000 dilution)	Sigma	Cat#A3854; RRID: AB_262011
Donkey Anti-Rabbit IgG, Whole Ab ECL Antibody, HRP Conjugated (1:2500 dilution)	GE Healthcare	Cat#NA934; RRID: AB_772206
Sheep Anti-Mouse IgG, Whole Ab ECL Antibody, HRP Conjugated (1:2500 dilution)	GE Healthcare	Cat#NA931; RRID: AB_772210

Donkey Anti-Rabbit Alexa Fluor® Plus 647 (1:2000 dilution)	ThermoFisher (Invitrogen)	Cat#A32795
Donkey Anti-Rabbit Alexa Fluor® Plus 488 (1:2000 dilution)	ThermoFisher (Invitrogen)	Cat#A32731
Donkey Anti-Mouse Alexa Fluor® Plus 594 (1:2000 dilution)	ThermoFisher (Invitrogen)	Cat#A11032
Donkey Anti-mouse Alexa Fluor® Plus 350 (1:2000 dilution)	ThermoFisher (Invitrogen)	Cat#A10035
Chemicals and Assay kits		
Glutathione (GSH,)	Millipore-Sigma	Cat#PHR1359
S-nitrosoglutathione (GSNO,),	Millipore-Sigma	Cat#N4148
N_{ω}-Nitro-L-arginine methyl ester (L-NAME,)	Millipore-Sigma	Cat#N5751
Dizocilpine **(MK-801,)**	Millipore-Sigma	**Cat#M107**
Glutaraldehyde solution	Millipore-Sigma	**Cat# G7651**
N-Methyl-D-aspartic acid (NMDA)	Millipore-Sigma	Cat# **M3262**
Adenosine 5′-triphosphate (ATP) disodium salt hydrate	Millipore-Sigma	Cat#A2383
Adenosine 5′-diphosphate (ADP) sodium salt	Millipore-Sigma	Cat#A2754
Adenosine 5′-[γ-thio]triphosphate (ATPγS) tetralithium salt	Millipore-Sigma	Cat#A1388
Neocuproine	Millipore-Sigma	Cat#N1501
EZ-Link™ HPDP-Biotin	ThermoFisher Scientific	Cat# 21341
1X PBS pH 7.4	Quality Biologicals	Cat#114-058-101
10X TBS pH 7.4	Quality Biologicals	Cat#351-086-101
Phosphatase inhibitor cocktail 2	Millipore-Sigma	Cat#P5726
Phosphatase inhibitor cocktail 3	Millipore-Sigma	Cat#P0044
Paraformaldehyde (PFA) reagent grade	Millipore-Sigma	Cat#P6148
Normal donkey serum	Jackson ImmunoResearch	Cat#017-000-121
pHrodo™ Red AM Intracellular pH Indicator	ThermoFisher Scientific	Cat# P35372
ADP Colorimetric Assay Kit	ThermoFisher Scientific	Cat#MAK081
PIERCE BCA PROTEIN ASSAY	ThermoFisher Scientific	Cat#23227
DreamTaq Green PCR Master Mix (2X)	ThermoFisher Scientific	Cat#K1082
Human insulin solution	Sigma	Cat#I9278
Rapamycin	LC laboratories	Cat#R-5000
Cell culture reagents		
DMEM - Dulbecco's Modified Eagle Medium	ThermoFisher Scientific	Cat#11965118
DMEM, no phenol red	ThermoFisher Scientific	Cat#31053028
FBS - fetal bovine serum	ThermoFisher Scientific	Cat#16000044
Penicillin-Streptomycin	ThermoFisher Scientific	Cat#15140122

Neurobasal™ Medium	ThermoFisher Scientific	Cat#21103049
B-27™ Plus Supplement (50X)	ThermoFisher Scientific	Cat#A3582801
Neurobasal™ Medium, minus phenol red	ThermoFisher Scientific	Cat#12348017
**Hank's balanced salt solution (HBSS)**	Millipore-Sigma	Cat#14025
CTS™ TrypLE™ Select Enzyme	ThermoFisher Scientific	Cat#A1285901
Lipofectamine 2000 Reagent	ThermoFisher Scientific	Cat#11668019
Experimental Models: Organisms/Strains		
Mouse: B6;129X1-ATAD1$^{1+/-}$	Zhang et al., 2011. Umanah et al., 2017	N/A
Mouse: C57BL/6J	Jackson Laboratory	JAX:000664
Oligonucleotides		
Thor-N-XhoI Fwd GATCTCGAGATGGTACATGCTGAAGCCTT TTCTCG	Integrated DNA Technologies, Inc. (*IDT*)	Oligo synthesis
Thor N10 Xho Fwd: GATCTCGAGATGTTGAGTCGGAACGAAGT TGTCGGTTTAA	IDT	Oligo synthesis
Thor N20 Xho Fwd: GATCTCGAGATGTTTCGTTTAACAATCTTT GGTGCAGTAAC	IDT	Oligo synthesis
Thor N30 Xho Fwd: GATCTCGAGATGTACTTCACTATTAAATGG ATGGTAGATGC	IDT	Oligo synthesis
Thor N40 Xho Fwd: GATCTCGAGATGATTGACCCCACCAGGAA GCAGAAAGTGG	IDT	Oligo synthesis
Thor N50 Xho Fwd: GATCTCGAGATGGCTCAGAAACAGGCGGA AAAACTG	IDT	Oligo synthesis
Thor N60 Xho Fwd: GATCTCGAGATGCAAATTGGTGTGAAAAAT GTGAAGC	IDT	Oligo synthesis
Thor N70 Xho Fwd: GATCTCGAGATGCAAGAGTATGAGATGAG TATTGCTGCTC	IDT	Oligo synthesis
Thor N80 Xho Fwd: GATCTCGAGATGCAAGTAGACCCTCTTAAT ATGCATGTTAC	IDT	Oligo synthesis
Thor N90 Xho Fwd: GATCTCGAGATGCAAAGTGATATAGCAGG TTTAGATGATG	IDT	Oligo synthesis
Thor N100 Xho Fwd: GATCTCGAGATGCAAACAGATCTGAAAGA CACAGTCATCC	IDT	Oligo synthesis
Thor C20 BamHI-Rev: ATCGGATCCATCCTCAATTGCCCGATGCA AGTCCTGCTG	IDT	Oligo synthesis

Thor-C-BamH1 Rev: ATCGGATCCATCATCTAAACAAACGTGAGTCAGAACGC	IDT	Oligo synthesis
mTORFL-Fwd: CATGGATCCACTATGCTTGGGACGGGTCCTGCCGTGGCCACCGCCAGTG	IDT	Oligo synthesis
mTORFL-Rev: AGTCTCGAGATGTTACCAGAAGGGACACCAGCCAATGTAGCACTGGCAG	IDT	Oligo synthesis
Recombinant Plasmid DNA		
AAV-CAG-GFP	Addgene	Cat#28014
pET32a	EMD Biosciences	Cat#69015-3
pGEX6P1	Amersham	Cat#27-4597-01
pThorase-AAV-GFP	Umanah et al., 2017	N/A
pThorase-GEX (GST)	Umanah et al., 2017	N/A
pThorase-RFP	This Manuscript.	N/A
pThorase-GEX (GST) variants	This Manuscript.	N/A
pmTOR-GEX (GST) full length	This manuscript	N/A
pLamp1-RFP	Addgene	Cat#1817
pLamp1-mGFP	Addgene	Cat#34831
peYFP-C1-mTOR	Addgene	Cat#73384
pRK5-HA-mCherry-Raptor	Addgene	Cat#73386
pMito-RFP	Chen, Umanah, et al., 2014	N/A
Deposited Data		
Raw data used for this study	This Manuscript	
Software and Algorithms		
ImageJ	NIH	https://imagej.nih.gov/ij/
Prism 6	GraphPad software	https://www.graphpad.com/scientific-software/prism/
ZEN lite	Zeiss	https://www.zeiss.com/microscopy/us/products/microscope-software/zen.html
PyMOL 3D structure viewer	Schrödinger	http://pymolwiki.org
Other		
RIPA Lysis and Extraction Buffer	Thermo Scientific	Cat#89901
E. coli BL21(DE3) RIL cells	Agilent Technologies	Cat#230240
2X Lammli Sample Buffer	Bio-Rad	Cat#1610737
Novex WedgeWell 8-16% Tris-Glycine Mini Gels	Thermo Scientific	Cat#XP08165BOX
SUPERSIGNAL WEST PICO PLUS	Thermo Scientific	Cat#34580
Blotting-Grade Blocker, nonfat dry milk	Bio-Rad	Cat#1706404
Restore Western blot stripping buffer	Thermo Scientific	Cat#21059
Nitrocellulose Membrane	Bio-Rad	Cat#1620115
Hamilton® GASTIGHT® syringe, 1700 series	Sigma	Cat#20972
Pierce High-Capacity Endotoxin Removal Spin Column	Thermo Scientific	Cat#88276

Superdex 200 Increase 10/300 G	GE Healthcare	Cat#45-002-570
Ni Sepharose 6 Fast Flow	GE Healthcare	Cat#17-5318-06
Prescission Protease	GE Healthcare	Cat#27-0843-01
ATPγS, Adenosine 5'-(3-thiotriphosphate) tetralithium salt	Millipore-Sigma	Cat#A1388-25MG
