# Supplementary material for: AAA + ATPase Thorase inhibits mTOR signaling through the disassembly of the mTOR complex 1
Source: Nat Commun. 2022 Aug 17;13:4836. doi: 10.1038/s41467-022-32365-2 (PMC9385847; doi:10.1038/s41467-022-32365-2)
Supplement: Supplementary file 2 — Reporting Summary [file 41467_2022_32365_MOESM2_ESM.pdf]

nature portfolio

Corresponding author(s): NCOMMS-22-14595-T

Last updated by author(s): Jun 8, 2022

Reporting Summary

Nature Portfolio wishes to improve the reproducibility of the work that we publish. This form provides structure for consistency and transparency in reporting. For further information on Nature Portfolio policies, see our Editorial Policies and the Editorial Policy Checklist.

Statistics

For all statistical analyses, confirm that the following items are present in the figure legend, table legend, main text, or Methods section.

n/a	Confirmed	
☐	☒	The exact sample size (*n*) for each experimental group/condition, given as a discrete number and unit of measurement
☐	☒	A statement on whether measurements were taken from distinct samples or whether the same sample was measured repeatedly
☐	☒	The statistical test(s) used AND whether they are one- or two-sided<br>*Only common tests should be described solely by name; describe more complex techniques in the Methods section.*
☐	☒	A description of all covariates tested
☐	☒	A description of any assumptions or corrections, such as tests of normality and adjustment for multiple comparisons
☐	☒	A full description of the statistical parameters including central tendency (e.g. means) or other basic estimates (e.g. regression coefficient) AND variation (e.g. standard deviation) or associated estimates of uncertainty (e.g. confidence intervals)
☐	☒	For null hypothesis testing, the test statistic (e.g. *F*, *t*, *r*) with confidence intervals, effect sizes, degrees of freedom and *P* value noted<br>*Give P values as exact values whenever suitable.*
☒	☐	For Bayesian analysis, information on the choice of priors and Markov chain Monte Carlo settings
☒	☐	For hierarchical and complex designs, identification of the appropriate level for tests and full reporting of outcomes
☐	☒	Estimates of effect sizes (e.g. Cohen's *d*, Pearson's *r*), indicating how they were calculated

Our web collection on statistics for biologists contains articles on many of the points above.

Software and code

Policy information about availability of computer code

Data collection	ZEN lite (Zeiss)
Data analysis	ImageJ (NIH); Prism 9 (Graph Pad software)

For manuscripts utilizing custom algorithms or software that are central to the research but not yet described in published literature, software must be made available to editors and reviewers. We strongly encourage code deposition in a community repository (e.g. GitHub). See the Nature Portfolio guidelines for submitting code & software for further information.

Data

Policy information about availability of data

All manuscripts must include a data availability statement. This statement should provide the following information, where applicable:

- Accession codes, unique identifiers, or web links for publicly available datasets
- A description of any restrictions on data availability
- For clinical datasets or third party data, please ensure that the statement adheres to our policy

Mass spectrometry data generated for this study is available at the Harvard Medical School Taplin Spectrometry Facility database upon request. Additional raw data generated for this manuscript will be made publicly available through a public data repository by the time of publication.

Human research participants

Policy information about studies involving human research participants and Sex and Gender in Research.

Reporting on sex and gender	n/a
Population characteristics	*Describe the covariate-relevant population characteristics of the human research participants (e.g. age, genotypic information, past and current diagnosis and treatment categories). If you filled out the behavioural & social sciences study design questions and have nothing to add here, write "See above."*
Recruitment	*Describe how participants were recruited. Outline any potential self-selection bias or other biases that may be present and how these are likely to impact results.*
Ethics oversight	*Identify the organization(s) that approved the study protocol.*

Note that full information on the approval of the study protocol must also be provided in the manuscript.

Field-specific reporting

Please select the one below that is the best fit for your research. If you are not sure, read the appropriate sections before making your selection.

☒ Life sciences ☐ Behavioural & social sciences ☐ Ecological, evolutionary & environmental sciences

For a reference copy of the document with all sections, see nature.com/documents/nr-reporting-summary-flat.pdf

Life sciences study design

All studies must disclose on these points even when the disclosure is negative.

Sample size	All biochemical and imaging experiments were repeated independently at least three times. For biochemical experiments involving brain tissue or mouse embryonic fibroblasts, 3 biological replicates per group were used per experiment. For mouse survival experiments and immunohistochemical analysis, enough sample size (as determined via post hoc power analysis) was used, and mice pertaining to different litters were used.
Data exclusions	n/a
Replication	Results were reproducible when experiments were performed by 2 independent researchers
Randomization	Sample randomization for biochemical experiments at least 2 independent researchers, and at least 3 biological replicates total per group were used. For mouse experiments where either vehicle or rapamycin was injected, an independent researcher randomly allocated mice to either treatment groups.
Blinding	During data collection, for immunofluorescence, immunohistochemistry and electron microscopy imaging, researchers were blinded to genotypes. For mouse survival analysis, researchers were blinded to both genotype and treatment.

Reporting for specific materials, systems and methods

We require information from authors about some types of materials, experimental systems and methods used in many studies. Here, indicate whether each material, system or method listed is relevant to your study. If you are not sure if a list item applies to your research, read the appropriate section before selecting a response.

Materials & experimental systems

n/a	Involved in the study	
☐	☒	Antibodies
☐	☒	Eukaryotic cell lines
☒	☐	Palaeontology and archaeology
☐	☒	Animals and other organisms
☒	☐	Clinical data
☒	☐	Dual use research of concern

Methods

n/a	Involved in the study	
☒	☐	ChIP-seq
☒	☐	Flow cytometry
☒	☐	MRI-based neuroimaging

Antibodies

Antibodies used	Mouse monoclonal anti-Thorase, NeuroMab, Cat#75-157<br>Rabbit monoclonal anti-mTOR, Cell Signaling Technologies, Cat#2983<br>Rabbit monoclonal anti-phos-S2448 mTOR, Cell Signaling Technologies, Cat#5536<br>Rabbit monoclonal anti-phos-S2481 mTOR, Cell Signaling Technologies, Cat#2974<br>Rabbit monoclonal anti-Raptor, Cell Signaling Technologies, Cat#2280 Rabbit monoclonal anti-phos-S792 Raptor, Cell Signaling Technologies, Cat#2083<br>Rabbit monoclonal anti-Rictor, Cell Signaling Technologies, Cat#2114 Rabbit monoclonal anti-p70 S6K, Cell Signaling Technologies, Cat#2708 Rabbit monoclonal anti-phos-T389 p70 S6K, Cell Signaling Technologies, Cat#9205<br>Rabbit monoclonal anti-S6, Cell Signaling Technologies, Cat#2217 Rabbit monoclonal anti-phos-S240/244 S6, Cell Signaling Technologies, Cat#2215<br>Rabbit monoclonal anti-4EBP1, Cell Signaling Technologies, Cat#9644 Rabbit monoclonal anti-phos-T37/46 4EBP1, Cell Signaling Technologies, Cat#2855<br>Rabbit monoclonal anti-elF4B, Cell Signaling Technologies, Cat#3592 Rabbit monoclonal anti-ULK1, Cell Signaling Technologies, Cat#8054<br>Rabbit monoclonal anti- phos-S757 ULK1, Cell Signaling Technologies, Cat#6888<br>Rabbit monoclonal anti-Rheb1, Cell Signaling Technologies, Cat#13879<br>Anti-rabbit IgG-HRP conjugate secondary, Abcam, Cat# ab98467;<br>Anti-mouse IgG-HRP conjugate secondary, Abcam, Cat# ab6823<br>Mouse anti-β-actin-HRP, Sigma, Cat#A3854<br>Donkey Anti-Rabbit IgG, Whole Ab ECL Antibody, HRP Conjugated, Cat#NA934 Sheep Anti-Mouse IgG, Whole Ab ECL Antibody, HRP Conjugated, Cat#NA931<br>Donkey Anti-Rabbit Alexa Fluor® Plus 647, ThermoFisher, Cat#A32795<br>Donkey Anti-Rabbit Alexa Fluor® Plus 488, ThermoFisher, Cat#A32731<br>Donkey Anti-Mouse Alexa Fluor® Plus 594, ThermoFisher, Cat#A11032<br>Donkey Anti-mouse Alexa Fluor® Plus 350, ThermoFisher, Cat#A10035
Validation	*Describe the validation of each primary antibody for the species and application, noting any validation statements on the manufacturer's website, relevant citations, antibody profiles in online databases, or data provided in the manuscript.*

Eukaryotic cell lines

Policy information about cell lines and Sex and Gender in Research

Cell line source(s)	HEK293FT (ThermoFisher Scientific; Cat# R700-07) , MEFs (Mouse Embryonic Fibroblasts); TSC2 KO cell lines; RagA GTP/GTP cell lines
Authentication	Cell line was used and maintained following the company procedure
Mycoplasma contamination	Mycoplasa contamination negative
Commonly misidentified lines<br>(See ICLAC register)	n/a

Animals and other research organisms

Policy information about studies involving animals; ARRIVE guidelines recommended for reporting animal research, and Sex and Gender in Research

Laboratory animals	Thorase-heterozygous C57BL/6J mice (ATAD1+/–)
Wild animals	n/a
Reporting on sex	n/a
Field-collected samples	n/a
Ethics oversight	Followed the guidelines of Laboratory Animal Manual of the National Institute of Health Guide to the Care and Use of Animals and upon approval of the Johns Hopkins Medical Institute Animal Care and Use Committee.

Note that full information on the approval of the study protocol must also be provided in the manuscript.
